# Impacts of Social and Emotional Learning Interventions for Teachers on Teachers' Outcomes: A Systematic Review With Meta-Analysis

**DOI:** 10.3389/fpsyg.2021.677217

**Published:** 2021-07-01

**Authors:** Sofia Oliveira, Magda Sofia Roberto, Nádia Salgado Pereira, Alexandra Marques-Pinto, Ana Margarida Veiga-Simão

**Affiliations:** Centro de Investigação em Ciência Psicológica, Faculdade de Psicologia, Universidade de Lisboa, Lisboa, Portugal

**Keywords:** intervention, meta-analysis, professional development, social and emotional learning, systematic review, teachers

## Abstract

Teaching is among the most emotionally demanding jobs, impacting teachers' personal lives and job performance. Since teaching-specific stressors are mainly socio-emotional related, social and emotional learning (SEL) interventions targeting teachers have increased rapidly in recent years. This study conducted a systematic review with meta-analysis of 43 empirical studies which evaluated the efficacy of school-based SEL interventions involving 3,004 in-service preK-12 teachers. The initial systematic review showed that these interventions were very heterogeneous and the research on their efficacy assessed widely distinct outcome variables. Concerning the meta-analysis, results showed statistically significant small to medium effect sizes favoring the experimental group, with SEL interventions impacting teachers' social and emotional competence [*g* = 0.59, 95% CI (0.29, 0.90)], well-being [*g* = 0.35, 95% CI (0.16, 0.54)], and psychological distress [*g* = −0.34, 95% CI (−0.57, −0.10)]. Meta-regressions did not reveal significant values of the explanatory variables, and publication bias was found for social and emotional competence and well-being domains. Findings add to growing empirical evidence regarding the impact of these interventions and contribute to the development of guidelines for the design of effective SEL interventions for teachers.

## Introduction

Over the last decade, education and mental health have been referred to as social, political and scientific priority issues requiring attention, and schools have been acknowledged as the primary context in which equity in young people's access to quality learning and developmental opportunities may be enhanced (UNESCO, 2018). Thus, teachers are expected to actively respond to both students' academic and social and emotional needs (e.g., Jennings and Greenberg, 2009). However, teachers' initial training focuses mainly on the academic domain, while they lack explicit training as regards the Social and Emotional Competence (SEC) domain. This absence of training is mainly in terms of intra-personal competences such as being able to identify and adequately manage their emotions and behaviors, and to monitor their own progress toward achieving goals [for an extensive review on how SEC development is integrated in teacher preparation programs across the USA see Schonert-Reichl et al. (2017)], which appears to influence not only their own well-being but also students' achievement and behavior (Crain et al., 2017; Schonert-Reichl, 2017).

Not surprisingly, teaching has been described as an emotionally demanding job linked to frequent episodes of work-related stress and burnout (Jennings and Greenberg, 2009; Marques-Pinto and Alvarez, 2016). Hence, the teaching profession presents particular risks as far as teachers' occupational health is concerned, affecting not only their mental health and well-being but also classroom management and instructional practices which, in turn, affect students' engagement and academic achievement (e.g., Jennings and Greenberg, 2009; Durlak et al., 2015; Schonert-Reichl, 2017).

Therefore, efforts have been made to identify and enhance protective factors that may act as a buffer against occupational stress and burnout caused by the challenges of teaching (Durlak et al., 2015). In this scenario, the promotion of social and emotional competencies has emerged in the literature as one of the main protective factors from which teachers can particularly benefit since they are crucial to classroom management and classroom climate, two key features of teaching efficacy, leading to an increase in teachers' job performance (Jennings and Greenberg, 2009).

As a result, Social and Emotional Learning (SEL) interventions seeking to directly promote teachers' SEC have increased over the last decade (Jennings et al., 2017; Schonert-Reichl, 2017). Nonetheless, these interventions are, to date, highly distinct regarding their approach, content, format and dosage (Wigelsworth et al., 2016). Furthermore, research on their efficacy is scarce and has tended to focus more on an assessment of diverse outcome domains, thus, limiting the comparison and overview of these interventions (Jennings et al., 2017) and the establishment of guidelines for the development of effective SEL interventions for teachers. In fact, most of the literature on SEL interventions within educational contexts has emphasized student- and classroom-level outcomes (Domitrovich et al., 2016; Greenberg and Abenavoli, 2017). Only recently have evidence-based studies on how SEL interventions targeting teachers' impact on teacher-level outcomes begun to emerge steadily on a worldwide scale, pointing to promising results (e.g., Harris et al., 2016; Carvalho, et al., 2017; Castillo-Gualda et al., 2017; Jennings et al., 2017).

Nonetheless, the professional development of teachers has gained momentum over the last decades, and several guidelines highlighting the role of variables, such as the dosage of intervention, cross-session training, and the specific nature of the contents addressed in the development of effective interventions for teachers in general, have emerged (Gulamhussein, 2013). Additionally, the literature has also given prominence to several consensual standards for identifying the best empirically supported interventions, such as the use of experimental designs with participants' random assignment to treatment groups, the use of follow-up measures, and independent research trials (Biglan et al., 2003) and the control of biases (Higgins et al., 2011). However, the suitability and relevance of these guiding references have yet to be studied when specifically applied to SEL interventions for teachers.

### A Brief History of the SEL Rationale

Twenty-five years ago, with the forthcoming twenty-first century, an expansion of sociopolitical norms on academic success and quality education to include non-academic skills was seen (Osher et al., 2016). Thus, schools became flooded with a myriad of interventions aiming to prepare children and youths to face future challenges (Durlak et al., 2015). These interventions, mostly based on the Positive Youth Development movement were, however, developed in a splintered and uncoordinated manner (Elias et al., 1997). Nonetheless, despite targeting different and apparently non-related behaviors (e.g., career education, sex education, violence prevention, health education, and nutrition education), these interventions shared a common basis established within a set of cross-cutting social, emotional, and behavioral skills (Greenberg et al., 2003). Hence, in 1997, Elias et al. first introduced and defined the SEL rationale with a view to creating a regulatory board for the centralization and standardization of intervention and evaluation policies and practices seeking to promote the optimal development of children and youths. In this scenario, the SEL rationale emerged with the purpose of establishing a common framework to systematize, guide and assess student-targeting interventions which were proliferating within schools at the end of the twentieth century, in order to optimize their contributions (Durlak et al., 2015). Thus, the SEL rationale results from a need to operationalize constructs and it is not presented as a theoretical framework for the practice, therefore it has been referred to as atheoretical (Tolan et al., 2016).

Nevertheless, the original authors identified some theoretical frameworks as primarily informant sources to which practitioners and researchers should resort for the design and implementation of their intervention programs (Durlak et al., 2015). However, when consulting these sources, the goal of developing competences in youths that promote their optimal adaptation to life challenges (Elias et al., 1997) should always be at the forefront. The following theoretical frameworks have been recommended to help program developers and researchers create and evaluate SEL interventions: systems theories, theories on emotional intelligence, social development and social skills training, and theories related to development, learning, and behavior change (Durlak et al., 2015; Osher et al., 2016; Tolan et al., 2016). When designing or evaluating an intervention, these theoretical frameworks should be taken into consideration to inform: (1) what to change (i.e., what specific contents should be included within the program; e.g., Emotional intelligence theory); (2) how to change (i.e., specific strategies through which the program should promote the change; e.g., Social cognitive theory, Social information-processing theory); (3) where / with whom to change (i.e., in what context and / or with whom; e.g., Ecological systems theory; Durlak et al., 2015; Tolan et al., 2016). Hence, the SEL rationale, in essence, results from multiple and isolated lines of empirical research which have been driven from different theoretical frameworks that have not always clear and distinct boundaries (Tolan et al., 2016).

Nowadays, SEL is defined as the process by which individuals acquire and apply core skills in five interrelated areas i.e., self and social-awareness, self-management, relationship skills, and responsible decision making, referred to as SEC (Durlak et al., 2015). Despite its practice-centered origin, two theoretical frameworks mostly inspired the conceptualization and operationalization of the SEL areas. On the one hand, the Emotional intelligence theory (Salovey and Mayer, 1990) inspired the development of the emotional-related areas. On the other hand, the social skills training movement, based on Bandura's social learning theory (Bandura, 1969), appears to have inspired the development of the self-regulation and interpersonal relationship areas (Marques-Pinto and Raimundo, 2016). Understandably, the SEL rationale shares with other coexisting frameworks the purpose of promoting optimal development (e.g., the Social Competence rationale) and the same underlying conceptualizations of social and emotional functioning, thus making the clear distinction between SEC and other categories of psychological functioning a challenge (Tolan et al., 2016). However, the SEL rationale may be distinguished from other approaches that share the same main goal, such as the Positive Youth Development and the Positive Psychology rationales which rely on clearly distinct conceptual frameworks (e.g., Developmental systems, Humanistic psychology; Tolan et al., 2016).

According to the SEL rationale as known today, SEL interventions are considered to be those which aim to promote SEC, through the explicit instruction of these intra- and interpersonal core skills, and based on a learner-centered learning approach (Durlak et al., 2015; Tolan et al., 2016). It is by means of this learner-centered approach that individuals become capable of identifying and regulating emotions, establishing and pursuing positive goals, appreciating, establishing and maintaining healthy relationships, making ethical, social and personal, responsible decisions, and of managing situations positively (Durlak et al., 2015). Considering this rationale, SEL is based on the idea that the acquisition of SEC occurs within social contexts through the relationships one establishes with others, but also through how each individual responds subjectively to these interpersonal experiences (Durlak et al., 2015). Additionally, the SEL rationale consistently states that SEC will act as a protection factor (Durlak et al., 2015). This approach assumes that SEC are an asset to which one may resort in order to better respond to potential risk situations, however the involvement in risk behaviors is not only predicted by the presence / absence of SEC but also by a set of different factors that should be taken into account (e.g., the context; Tolan et al., 2016). Additionally, it stems from the belief that SEC may be learned, trained and developed through a learner-centered and explicit teaching approach. Therefore, in this non-dispositional and non-dichotomous orientation, the SEL rationale is viewed as being detached from other movements such as for instance, Positive Youth Development and Positive Psychology. Indeed, while the latter share the common goal of promoting children and youths' optimal development, there is also divergence within these two orientations (Tolan et al., 2016).

This practice-centered approach, where multiple theoretical frameworks can inform the same program, coupled with the coexistence of other rationales that share a common goal, makes it difficult to establish a clear definition of the SEL rationale's frontiers. Furthermore, cultural appropriations of the SEL definition have served to increase this complexity (Cefai et al., 2018) and lead to the concurrence of multiple languages regarding the same construct (Greenberg et al., 2003; Humphrey et al., 2011; Jones and Bouffard, 2012). This simultaneity of different yet similar languages is mirrored by the different consortia of SEL worldwide (Durlak et al., 2015). With the constitution of the SEL rationale (i.e., Elias et al., 1997), a consortium was created in the US which, to date, is the most frequently mentioned when referring to SEL, namely the Collaborative for Academic, Social, and Emotional Learning (2020). However, as research on SEL interventions' impact on children and youths' social, emotional, and academic competencies began to increase and consolidate (e.g., Durlak et al., 2010, 2011; Sklad et al., 2012; Corcoran et al., 2018), other organizations emerged seeking to contribute to a global, yet culturally adjusted dissemination of the SEL rationale (Durlak et al., 2015; Cefai et al., 2018). Some examples of these other consortia are the European Network for Social and Emotional Competence (ENSEC, 2019) in Europe, KidsMatter and MindMatters frameworks in Australia (Australian Government, 2020), the Social and Emotional Aspects of Learning (SEAL) program in the UK (UK Government, 2010), and the Wallace Foundation (2020) in the US. Despite the diversity of terms used to refer to the same construct (e.g., Social and Emotional Learning vs. Social and Emotional Education, Social and Emotional Skills vs. Social and Emotional Competence), all of these consortia have highlighted the same five key-competencies (Cefai et al., 2018). Additionally, different terms, namely Social and Emotional Well-being, Non-cognitive skills, Soft skills, have been associated with the SEL rationale, although they refer to distinct specific competencies (e.g., flexibility) which emerge within other fields of study (e.g., mental health, neurosciences, vocational, and career) (Cefai et al., 2018).

As a result of the aforementioned issues, the literature on SEL has faced serious concerns regarding inconsistencies in operationalization processes, definition, and measurement (Humphrey et al., 2011). Furthermore, although some meta-analyses of reference in the area have made the distinction between rationales (e.g., Positive Youth Development and SEL), they have not clarified / discussed the underlying psychological theories of social emotional learning that are pertinent to the analyzed studies (e.g., Taylor et al., 2017), or justified the search terms used (e.g., Sklad et al., 2012). In addition, when said analysis were conducted, all the search terms were mixed (e.g., Durlak et al., 2011), thus compromising the reliability and validity of the findings.

Table 1 presents a synthesis of: (1) the various rationales that have coexisted with SEL for the promotion of children and youths' optimal development since the end of the twentieth century; (2) the multiple strategies that have been used for the promotion of SEC, within the SEL rationale; (3) the multiple terms that have been used to refer to SEL and SEC across the different consortia. A more in-depth analysis on the different common terms and rationales may be found in Cefai et al. (2018).

**Table 1:** Synthesis of the multiple dimensions that increase the complexity of identifying and bordering the SEL rationale [based on the works of Elias et al. (1997), Durlak et al. (2015), Tolan et al. (2016), and Cefai et al. (2018)].

**Examples of…**	
… rationales seeking to promote children and youths' optimal development	Affective Education, Character Education, Citizenship / Civic Education, Deeper Learning, Emotional Intelligence, Health Promotion, Life Skills Training, Personal and Social Development, Positive Psychology, Positive Youth Development, Social and Emotional Learning, Social Competence, twenty-first Century Skills
… strategies used within interventions for the promotion of SEC	Cognitive therapy, Cognitive and behavioral therapy (CBT), Coping skills training, Emotional intelligence training, Intentions to behave training, Mindfulness, Social learning through modeling and feedback, Social skills training
… common terms used to define and refer to SEL and SEC in the literature	Social and emotional learning, Social and emotional education, Social and emotional skills, Social and emotional competence, Social and emotional well-being, Soft skills, Non-cognitive skills

In short, SEL is not a conceptually driven theoretical understanding of SEC. Instead, it emerged as a subsuming overall framework for organizing many different preventive and promotive interventions, making the delimitation of clear boundaries between SEC and other psychological functioning categories a difficult endeavor (Tolan et al., 2016). Although several authors make reference to different theories that may inform SEL interventions regarding what to change, how to do so, and where / with whom, the field may be characterized as multiple isolated lines of empirical inquiry stemming from different theoretical frameworks, with unclear overlaps and distinctions (Tolan et al., 2016), thus lacking clarification and integration as reflected in prior research (e.g., Durlak et al., 2011; Sklad et al., 2012; Taylor et al., 2017).

Hence, for the purpose of this study, in an effort to guarantee homogeneity within the analyzed studies, SEL was operationalized as “the process through which children and adults develop the skills, attitudes, and values necessary to acquire social and emotional competence” (Elias et al., 1997, p. 2), with SEC being defined as the five key-competencies, which are common to all consortia for / approaches to SEL, i.e., self-awareness, self-management, social awareness, relationship skills, and responsible decision making (Durlak et al., 2015).

### From SEL for Children and Youths to SEL Interventions for In-service Teachers

Since the inception of SEL, its research and intervention have faced three main waves in the trajectory toward a one whole school approach (Greenberg et al., 2003; Osher et al., 2016). While initial papers on SEL referred only to the importance and contribution of the promotion of SEC for children and youths' development, a more systemic approach soon began to emerge with the role of teachers being recognized. However, only recently has SEL for teachers *per se* been considered (Jennings and Greenberg, 2009; Durlak et al., 2015; Schonert-Reichl, 2017), resulting from the acknowledgment that (1) teachers could enhance the impact of SEL on students if they explicitly infused SEL within their classrooms; (2) and that teachers lacked explicit training in SEL and, therefore, professional development training for teachers on how to teach SEL programs' specific content to their students was required (Greenberg et al., 2003; Osher et al., 2016). On the other hand, most SEL programs continued to assume that teachers were prepared to effectively act as a social and emotional competent role model almost in a dispositional manner (Greenberg et al., 2003; Jennings and Greenberg, 2009; Durlak et al., 2015; Marques et al., 2019). These assumptions may have delayed the establishment of a SEL line of intervention specifically targeting teachers and their own SEC development, which has only begun to be addressed in the last decade (Schonert-Reichl, 2017; Marques et al., 2019), thus explaining why student-centered approaches have continued to be the main focus of SEL over time.

When applied to a professional development context, SEL interventions are described as a set of practices and policies which enhance personal development, positive interpersonal relationships, in addition to effective and ethical work and performance (Durlak et al., 2015). Consequently, and mirroring the aforementioned observations concerning SEL for children and youths, when considering SEL for teachers the main issues regarding the non-theoretically driven framework underlying the conceptualization of the rationale appear to gain prominence. Once again, the background literature on the SEL rationale for teachers suggests that this approach is more an operational than a conceptually driven framework (e.g., Jennings and Greenberg, 2009; Durlak et al., 2015; Schonert-Reichl, 2017). Nevertheless, this issue is yet to be studied (e.g., Marques et al., 2019) and deserves further clarification. Moreover, along with SEL for children and youths, SEL for teachers maintains the Emotional intelligence theory as a main theoretical framework of reference, but is also primarily informed by the Transactional model of stress and coping (Lazarus and Folkman, 1984; providing information on main teacher-specific stressors and strategies for stress management) and the Self-determination theory (Deci and Ryan, 1985; providing information on teacher-specific needs which might directly relate to an increased perception of professional competence and of how to promote motivation for behavior change and learning) (Jennings and Greenberg, 2009). In this scenario, and adopting an isomorphic three-level model as previously presented, these theoretical frameworks may contribute to informing the development of interventions and research with regard to the first two levels, i.e., what to change (content level), and how to change (strategy level). At the content level, when referring to SEL for teachers, SEC mirror a specific set of social, emotional, and cognitive skills, as presented in Table 2.

**Table 2:** Description of teacher-specific social, emotional, and cognitive skills within each SEC [retrieved from Jennings and Greenberg (2009), p. 495].

**Domain**	**Specific skills**
Self and social awareness	To recognize and understand emotions and emotional patterns of their own and of others. To understand / be aware of how their emotional expressions affect their interactions with others. To have a realistic understanding of their abilities and recognize their emotional strengths and weaknesses. To be culturally sensitive and understand different perspectives. To motivate learning in themselves and others, though the promotion and use of emotions. To build strong and supportive relationships through mutual understanding and cooperation. To effectively negotiate solutions to conflict situations.
Self and relationship management	To manage their behavior even when emotionally aroused by challenging situations. To regulate their emotions in healthy ways that facilitate positive classroom outcomes without compromising their health. To effectively set limits firmly, yet respectfully. To be comfortable with a level of ambiguity and uncertainty that comes from letting students figure things out for themselves.
Responsible decision making	To display prosocial values and decide ethically, based on the assessment of factors such as the impact of their decisions on themselves and others. To respect others and take responsibility for their decisions and actions.

Additionally, up to now, SEL interventions have differed in, for example, their approaches, dosage, and the importance placed on each SEC-related area (Wigelsworth et al., 2016). Particularly, as regards SEL interventions for teachers, we may find interventions following universal approaches (i.e., in which the contents presented are geared toward all teachers, regardless of the grade-level they teach or their individual characteristics; e.g., Jennings et al., 2013), or targeting specific needs (e.g., the contents presented are grounded in the challenges elementary teachers face within their classroom; Murray et al., 2018). As far as dosage is concerned, these interventions are widely distinct, varying from short-term actions such as workshops (e.g., 2-h length; Wills et al., 2018) to medium/long-term approaches such as programs (e.g., 50-h length; Carvalho, et al., 2017). Finally, concerning SEC-related areas, some SEL interventions for teachers emphasize only a specific domain (e.g., Domitrovich et al., 2016), while others target all five areas (e.g., Cook et al., 2017). Thus, considering such variability, further knowledge of the factors that may influence the impact of these interventions on teachers is needed in order to establish guidelines which may lead to effective SEL interventions, thus guaranteeing high-quality implementation (Durlak et al., 2015; Schonert-Reichl, 2017).

Although recent, the literature on SEL interventions specifically developed for teachers has drawn attention owing to its positive impact on both teachers' personal and professional levels, and its contribution not only to teachers' well-being and performance, but also those of their students (Durlak et al., 2015; Schonert-Reichl, 2017). Firstly, research has suggested an impact on teachers' SEC, which has consisted specifically of outcomes that directly express one or more of the five key-competencies addressed by the SEL rationale, referring to particular expressions therein (e.g., emotional acknowledgment, emotional regulation, social competence, and self-regulation). Besides the direct and proximal effect of these interventions on the promotion of the SEC domain, a high degree of SEC among teachers has also been linked to a further four distal and indirect domains. On a personal level, greater SEC have been associated with a decrease in teachers' psychological distress, referring to outcomes regarding psychological discomfort or internalizing problems (e.g., negative affect, rumination, stress, anxiety and depression symptoms, emotional exhaustion, and depersonalization); and in teachers' physical distress, comprising outcomes associated with subjective health complaints, and behavioral and physiological health indicators (e.g., ache-related symptoms, insomnia, cortisol levels, blood pressure, respiratory, and heart rate; e.g., Jennings et al., 2013, 2017; Roeser et al., 2013; Harris et al., 2016). However, on a personal level, a higher degree of SEC has also been linked with an increase in teachers' well-being, which specifically refers to outcomes related to personal well-being and positive emotions (e.g., positive affect, self-efficacy, personal accomplishment, job and life satisfaction; e.g., Jennings et al., 2013; Domitrovich et al., 2016; Carvalho, et al., 2017; Crain et al., 2017). Taken together, teachers with high SEC appear to be more capable of managing their job demands and achieving higher levels of work and home life satisfaction (e.g., Talvio et al., 2013; Crain et al., 2017). On a professional level, SEL interventions seem to have a distal impact on teachers' ability to manage classrooms and respond to their emotional challenges, specifically by positively impacting the classroom climate and instructional practices domain, which involves outcomes related to teacher practices and classroom climate (e.g., emotional and instructional support, personalized teacher-student interactions, and classroom management), thus leading to higher quality learning environments (e.g., Hagelskamp et al., 2013; Morris et al., 2013; Hickey et al., 2017; Murray et al., 2018). Additionally, due to the co-regulative nature of classroom interactions, when teachers act with SEC, they may also foster the development of SEC among their students (Jennings and Greenberg, 2009) which, subsequently, may lead to higher levels of student well-being and academic achievement (Durlak et al., 2011; Sklad et al., 2012; Taylor et al., 2017; Corcoran et al., 2018). In short, SEL interventions for in-service teachers appear to play a key role by helping them regulate their own emotions and deal more proficiently with their job requirements, thus, promoting a healthier classroom climate and students' social, emotional and academic learning (Osher et al., 2016; Schonert-Reichl, 2017).

Thus, SEL interventions designed for teachers have been developed with promising results (e.g., Roeser et al., 2013; Crain et al., 2017; Jennings et al., 2017), gradually calling out for systematic and consistent literature overviews regarding the impacts of SEL interventions on teachers, more specifically on their outcomes. Some recent systematic reviews (e.g., Emerson et al., 2017; Hwang et al., 2017; Lomas et al., 2017) and a meta-analysis (Klingbeil and Renshaw, 2018) have discussed the impacts of mindfulness-based interventions, which have been presented as a powerful strategy through which SEL may be achieved, on teachers' psychological distress and well-being, and job performance. However, as previously discussed (Table 1), mindfulness-based interventions are not the only type of strategy available to promote SEC development. In fact, traditional techniques which did not resort to mindfulness (e.g., Cognitive therapy, CBT, Coping skills training, Emotional intelligence training), have also been used to promote SEC. Furthermore, mindfulness-based interventions may be used to develop the mindfulness competence *per se*, as a content, instead of targeting the development of SEC. Likewise, Carvalho and Queirós (2019) conducted a systematic review of 28 studies on the efficacy of stress management interventions for in-service teachers, however, not all stress management interventions can be considered SEL interventions. Additionally, to the best of our knowledge, only one literature review has been published to date concerning SEL interventions developed for in-service teachers (i.e., Marques et al., 2019). Nonetheless, this was a first approach to the topic with the sole aim of mapping the quantity and type of SEL interventions for in-service teachers available at the moment. Hence, systematic reviews or meta-analysis specifically addressing the impacts of SEL interventions developed for teachers on in-service teachers' personal and professional outcomes are needed.

Within the scope of meta-analytic studies, the aforementioned co-regulatory nature of classroom interactions should be taken into consideration. When referring to interventions for the professional development of teachers, the ultimate goal is always to promote a better educational climate and thus improved student-level outcomes (e.g., Freire et al., 2012). Therefore, even when SEL interventions for the development of teachers' SEC *per se* are developed, they can, sometimes, be developed as a sub-product of a more global intervention aiming to prepare teachers to intervene with students (i.e., teaching teachers to teach SEL to students, i.e., combined intervention targeting teachers and students' SEL; e.g., the 4Rs program; Brown et al., 2010). Hence, just as the SEC of teachers may indirectly affect students' SEC, well-being and performance, some literature has suggested that likewise, when students are more socially and emotionally competent, this may have an indirect effect on teachers' SEC, well-being and performance (e.g., Carvalho et al., 2021). Therefore, since combined interventions have different ultimate goals when compared to interventions specifically targeting teachers' SEC development and, since it is not possible at this point to isolate the direct and indirect effects of the combined interventions, which may increase the heterogeneity of the pool of data and compromise the results, for meta-analysis purposes, SEL interventions for in-service teachers effects should be estimated individually for the two types of interventions.

### The Present Study

The aim of the present study is to conduct a systematic review with a meta-analysis of empirical studies assessing the efficacy of SEL interventions for in-service preK-12 (i.e., from pre-kindergarden to grade 12) teachers on their personal and professional outcomes. Regarding the systematic review process, with a more exploratory and comprehensive end, two research questions were established:

*Q1. Did the pooled studies state the theoretical foundations underlying the design and implementation of their SEL interventions for teachers?*

*Q2. What quality indicators of empirical-evidence did the pooled studies consider when designing both the intervention and research?*

Additionally, and in light of the prior literature, the following hypotheses were also established:

*H1. SEL interventions for teachers will increase SEC, well-being and classroom climate and instructional practices, and decrease psychological and physical distress in teachers*.

*H2. SEL interventions for teachers' effects will be predicted by intervention dosage, cross-session training, and adequacy of content presented to teachers' teaching grade, such as the presence of (a) higher dosage, (b) cross-session training, and (c) contents adjusted to teachers' teaching grade will contribute to higher intervention effects*.

Moreover, integrated in the meta-analytic study, this study aims to test the temporal stability and sleeper effects of the SEL interventions for teachers, and to explore whether the use of mindfulness techniques to promote SEL is a predictor of these interventions' effect.

## Method

This study followed the Preferred Reporting Items for Systematic Reviews and Meta-Analyses (PRISMA) guidelines (Moher et al., 2015). Regarding ethical considerations, since public documents are the object of study there are no need for institutional review boards approval (Cooper and Dent, 2011). Nevertheless, ethical obligations of methodological rigor were ensured. Supplementary Material with greater detail on the methodological procedures adopted (i.e., detailed information on databases consulted, selected descriptors, eligibility criteria, initial search results and the data collection process, presentation of the funnel plots to analyze possible publication bias, and variables included in the coding process) is provided.

### Eligibility Criteria

In order to address our research questions with quality and consistency, the studies were required to present an empirical study (with quasi-experimental or experimental designs) on the efficacy of a SEL intervention for in-service preK-12 teachers in their personal and / or occupational outcomes. Thus, papers targeting university and / or pre-service teachers and those that did not access impacts on teacher-level variables were excluded. For the meta-analysis procedures to be possible, studies were only considered when sufficient information was reported to calculate the effect sizes of the interventions' impacts. Additionally, studies were included whenever the full-text version was available and published in Psychology or Educational peer-reviewed journals, after 1995, thus excluding papers published in non-peer reviewed journals and gray literature. No language constraints were applied.

### Search Strategy

We began by systematically screening empirical studies, published in Psychology or Education journals, available on EBSCOhost web, b-ON, SCOPUS and / or SciELO databases. In accordance with the eligibility criteria, the search (conducted in mid-2020) was narrowed to: articles, with full-text available, containing empirical work, and published in peer-reviewed journals since 1995.

The search across databases was carried out through advanced search options, by crossing sets of keywords such as teacher, social and emotional learning, training, intervention, and program effectiveness, in the title, abstract or subject terms. To improve the sensitivity of the search, synonyms, different spellings, and singular/plural forms, verb forms, adjectives of the terms used as descriptors were considered. Additionally, in order to focus the analysis, we opted to restrict the search to studies that did not include the descriptor student (and derivatives) in the title. Whenever possible, studies were screened through a Boolean search. In order to deepen the search and to assure saturation of data, a hand search of reference lists, consortia guides and organization websites was also conducted to identify any further studies available.

This global analysis resulted in 774 initial records. After the removal of duplicates through SRA Deduplicator (Rathbone et al., 2015; *n* = 113), the titles and abstracts of the 661 identified studies were screened in order to select all the items that met the eligibility criteria. At this stage, all the records presented the title and abstract written in English. As a result, based on title and abstract screening, 582 studies were excluded. Subsequently, the full-text version of the 79 remaining studies that met the aims of this review was examined in detail. Finally, a total of 43 records meeting all the selection criteria were considered for the systematic review. The remaining 36 studies were excluded due to one of the following reasons: for not addressing a SEL intervention (*n* = 14), not aiming to promote SEC in teachers (*n* = 6), not assessing SEL intervention effect (*n* = 2), targeting pre-service teachers (*n* = 4), being a qualitative study (*n* = 1) or a systematic revision / meta-analysis (*n* = 1), not being published in a peer-review journal (*n* = 1), or not presenting a control group (*n* = 7). The majority of these 43 studies were written in English (*n* = 40) and the remaining records were written in Spanish (*n* = 3). These 43 studies were then grouped, considering the purpose of the meta-analytic procedure, into a subsample of 27 studies (i.e., targeting only teachers) and a subsample of 16 studies (i.e., those presenting a combined SEL intervention).

### Study Coding

The 43 selected records were coded based on 25 criteria defined in accordance with PRISMA recommendations on data items (i.e., participants, intervention, comparisons, outcomes, and study design; Moher et al., 2015) and variables, highlighted in the literature as influencing the efficacy of interventions (e.g., dosage, facilitator, teaching grade; Gulamhussein, 2013) and quality of the research (e.g., randomization process; Higgins et al., 2011). The first author coded all the studies selected for the analysis. The interrater agreement (IRR) was then computed by calculating the percentage of agreement with two additional researchers with expertise in the SEL rationale, who used the criteria list to code 13 studies (i.e., 30.23%). To assess the IRR, since a fully crossed design was used (i.e., the three coders rated the same set of records), the intra-class correlation (ICC) was computed to evaluate the reliability regarding the metric criteria-variables (e.g., fidelity report), and the Kappa variant for three coders was computed to nominal criteria-variables (e.g., type of SEC assessed; Hallgren, 2012). An ICC of .96 was obtained, revealing an excellent IRR for the metric criteria-variables within the three coders (Hallgren, 2012). A mean Kappa of .64 was found for the nominal criteria-variables among the three coders, revealing substantial agreement (Hallgren, 2012). Prior to the data analysis, the three reviewers discussed the coding differences and the studies were re-coded, with all experts agreeing on the final code.

#### Outcomes

Outcomes were coded on the basis of the afore-mentioned five domains in Chapter 1.2, which were selected in accordance with the indications advanced in prior literature as to the main impact areas of SEL interventions (Jennings and Greenberg, 2009; Schonert-Reichl, 2017) and supported by previous research (e.g., Marques et al., 2019): SEC, Psychological distress, Physical distress, Well-being, and Classroom climate and instructional practices. The subgroup analysis was computed for two assessment points in time [i.e., time 2 (posttest) and follow-up], making it possible to test the temporal stability and sleeper effects of the SEL interventions for teachers.

#### Covariates

Prediction effects were tested for dosage of intervention, cross-session training, suitability of content presented to teachers' teaching grade, and use of mindfulness techniques. These variables were selected in accordance with Gulamhussein (2013), Higgins et al. (2011), Biglan et al.'s (2003) guidelines, and Klingbeil and Renshaw (2018) results. Dosage was re-coded as an ordinal variable with three levels: 1–14, 15–29, and 30 h or more. Cross-session training was coded as a dichotomous variable indicating the presence or absence of training between formal sessions. As regards suitability of content presented to teachers' teaching grade, this was re-coded as a nominal variable with three conditions considering whether the intervention addressed only class-level teachers (e.g., elementary school), only discipline-level teachers (e.g., high school), or both (considering a SEL intervention with non-specific to group characteristics). Use of mindfulness techniques to promote SEL was coded as a dichotomous variable indicating the use or not use of this strategy in the intervention.

### Data Analysis

As the aim of the present study was to evaluate the impact of SEL interventions developed for teachers on teachers' personal and/or occupational variables, for the data analysis we coded and analyzed: (1) studies using the same intervention but taking different cohorts into consideration (e.g., replication, cultural adaptations) as distinct interventions; (2) papers using the same intervention and the same cohort, but reporting effects on different outcomes at time 2 or follow-up as a single intervention. Therefore, a total of 39 interventions (presented in 43 studies) were considered for the systematic review. Then, a subsample of 25 interventions (presented in 27 studies) were eligible for the meta-analysis targeting only teachers outcomes; and a subsample of 14 interventions (presented in 16 studies) were considered for the meta-analysis targeting combined interventions' effects.

The pooled studies were included in a meta analytic random effects model, taking into account between-studies' heterogeneity. Hedges' *g*, as an unbiased standardized measure of effect, was estimated by retrieving the following information from the pooled studies: intervention and control group means, standard deviations and sample sizes. Whenever part of the previous information was not available, the standardized measure of effect was converted from *t* and *F* statistics. In addition to correcting Cohen's *d* for bias in small samples, Hedges' *g* makes it possible to estimate an effect based on different outcomes and metric scales by standardizing results across studies (e.g., Kline, 2004).

Anticipating high heterogeneity levels, effects were grouped according to the following dimensions: SEC, psychological distress, physical distress, well-being and classroom climate and instructional practices. Effects were targeted at time 2, but whenever possible, estimates were also provided for follow-up. Given that most of the studies reported several effects leading to within studies dependence, the random effects model was estimated using Robust Variance Estimation (RVE) with correction for small samples, allowing for intra-study correlation. The estimated effect was computed by allocating more weights to studies with smaller variance (Fisher and Tipton, 2015) and a sensitivity analysis performed to check if the computed effect changed according to different correlation values (Hedges et al., 2010).

The *I*^2^ statistic was computed to measure heterogeneity across the studies and the following cut-off values were used for interpretation: *I*^2^ < 50% suggesting low heterogeneity, 50–75% revealing moderate heterogeneity and >75% indicating high heterogeneity (Higgins et al., 2003). Prediction intervals for the estimated effects were computed to provide lower and upper bound values for future effects (Harrer and Ebert, 2018). Meta-regression models were fit to evaluate the role of the covariates on the estimated effects, namely intervention dosage, cross-session training, suitability of content presented to teachers' teaching grade, and use of mindfulness techniques. Estimates significance was provided when the 95% confidence interval (CI) did not include the 0.

Publication bias was assessed using sensitivity analysis following Vevea and Woods (2005) weight-function modeling, where a meta-analytic model adjusted for publication bias using *p*-value cut-points and a pre-specified vector of weights for each corresponding *p*-value is compared to an unadjusted model. For *p*-values below 0.05 all effect sizes survive selection, with the chance of survival dropping for *p*-values higher than 0.05. A pattern suggesting publication bias occurs when the estimated effect size decreases from the unadjusted to the adjusted model. The following intervals (and weights) were used: <0.001 (1), 0.001 < 0.01 (1), 0.01 < 0.05 (1), 0.05 < 0.10 (0.8), 0.10 < 0.20 (0.7), 0.20 < 0.30 (0.6), and 0.30 < 1 (0.5) (Coburn, 2018; Coburn and Vevea, 2019).

Effects were converted to Hedges' *g* using the package esc (Lüdecke, 2019). We used robusta (Fisher et al., 2017), meta (Schwarzer, 2007), metafor (Viechtbauer, 2010), and weightr (Coburn and Vevea, 2019) packages designed for R environment (R Core Team, 2018) to perform all meta-analytic analyses.

## Results

### Descriptive Synthesis of the Selected Studies

The total sample comprised 39 interventions involving 3,004 in-service preK-12 teachers. A summary of general publication features can be found in Table 3 and the full data concerning all the criteria analyzed may be found in the Supplementary Tables 4.1–4.3. As regards the publication dates, the first empirical studies [with a (quasi-)experimental design] concerning on evaluation of efficacy of a SEL intervention for in-service teachers on teachers' outcomes were published in 2008 (i.e., Raver et al., 2008; Webster-Stratton et al., 2008). However, these studies were still addressing combined interventions, with the professional development of teachers emerging as a sub-product of a global intervention. In our pool, the first study which addressed an individual SEL intervention (i.e., only targeting teachers) was not published until 2010 (i.e., Delgado et al., 2010), 15 years after the establishment of the SEL rationale. The majority of the 39 interventions assessed have been published in the last 6 years (2015–2020; 51.28%).

**Table 3:** Report on general characteristics of the 39 reviewed interventions.

**Characteristics**	***N***	**%**
**Publication date**		
1995–2004	0	0.00
2005–2014	19	48.72
2015–2020	20	51.28
**Intervention features**		
Geographic area		
Asia	3	7.69
Europe	12	30.77
North America	24	61.54
School area		
Urban	15	38.46
Suburban	2	5.13
Semi-rural	1	2.56
Rural	0	0.00
Combination	6	15.38
Not reported	15	38.46
Target		
Only teachers	25	64.10
Teachers and students	14	35.90
Grade participants taught		
Class-level	19	48.72
Discipline-level	7	17.95
Combined	10	25.64
Not reported	3	7.69
State conceptual framework		
Yes	14	35.90
No	25	64.10
Dosage of intervention		
1–14 h	6	15.38
15–29 h	14	35.90
30 or more hours	19	48.72
Cross-session training		
Yes	16	41.03
No	23	58.97
**Methodological features**		
Independent research		
Yes	16	41.03
No	23	58.97
Intervention led by its author		
Yes	20	51.28
No	19	48.72
Randomization (control for selection bias)		
Participant-level	18	46.15
School-level	10	25.64
None	11	28.21
Blinding of participants and researchers (control for performance bias)		
Yes	2	5.13
No	22	56.41
Not specified	15	38.46
Blinding of outcome assessment (control for detection bias)		
Yes	13	33.33
No	6	15.38
Not specified	20	51.28
Incomplete outcome assessment (control for attrition bias)		
Yes	17	43.59
No	1	2.56
Not specified	21	53.85
Selective report (control for reporting bias)		
Yes	0	0.00
No	1	2.56
Not applicable	38	97.44
Fidelity report		
Yes	9	23.08
No	30	76.92
Time of assessment		
Pre-posttest	29	74.36
Pre-posttest and follow-up	10	25.64
Type of measures		
Self-report	31	79.49
Behavioral	3	7.69
Physiological	7	17.95
External observation	14	35.90
Outcomes assessed		
SEC	20	51.28
Psychological distress	23	58.97
Physical distress	11	28.21
Well-being	24	61.54
Classroom climate and instructional practices	17	43.59

Most interventions were conducted in North America (61.54%) and Europe (30.77%). All eligible interventions were school-based interventions with a universal approach (i.e., preventive interventions targeting all teachers). Since the interventions considered were developed in educational contexts, an analysis of the school's area was conducted. Some of the studies (38.46%) did not report information concerning school area characteristics. Of those reporting this data, 15 interventions were conducted within urban areas, while six took place in combined areas (e.g., urban and sub-urban areas).

Additionally, the majority of interventions targeted only teachers' SEL (64.10%) of pre- and elementary school levels (48.72%), with a mean age of 40.55 years (*SD* = 4.89) and 11.20 years of professional experience (*SD* = 3.67). Regarding their content, most interventions addressed at least two SEC-related areas (89.74%). More specifically, 28 interventions addressed self-awareness, 28 intervened in self-management, 28 involved social-awareness, 27 considered relationship skills, and 10 focused on responsible decision making. Only four interventions targeted merely one SEC-related domain and the key-competence addressed across all the interventions was Relationship Skills. All these four interventions were combined interventions.

With regards to Q1, most of the studies did not state the conceptual framework underlying the development of the SEL intervention used (64.10%). When referring to the quality indicators considered by the pooled studies (i.e., Q2) regarding the intervention features, most interventions were delivered to the participants by a facilitator integrated in the original intervention development team (51.28%). Moreover, most of the interventions targeted class-level teachers, 25.64% of the interventions did not ground the addressed topics specifically to teachers' teaching grade. The length of these 39 interventions ranged from 2 to 50 h (*M* = 26.36, *SD* = 10.14, *Mdn* = 28.00), although most of them lasted more than 14 h (84.62%) [thumb rule proposed by (Gulamhussein, 2013)]. Additionally, most of interventions (58.97%) did not include assignments and/or monitoring activities between formal sessions (e.g., homework assignments, tutoring sessions, and ongoing coaching). Regarding the strategy used, in these 39 interventions, 22 used traditional techniques to promote SEL (e.g., CBT; 56.41%). The remaining 17 interventions (43.59%) resorted to the use of mindfulness techniques to increase SEL, of which 16 only targeted teachers and one intervention was a combined SEL intervention (i.e., Carvalho, et al., 2017). Some interventions were evaluated in more than one study, namely BEST in CLASS (*n* = 2), CARE (*n* = 2), Incredible Years—Teacher Classroom Management (*n* = 5), adapted MBSR (*n* = 4), RULER (*n* = 3), and SMART-in-Education (*n* = 3).

As regards the methodological features of the research design, there were 28 randomized-controlled trials [18 with a teacher-level randomization (46.15%) and 10 with a cluster-level randomization (25.64%)] suggesting low risk of selection bias (Higgins et al., 2011), 16 studies led by independent research teams (41.03%), and nine studies reporting fidelity levels (23.08%). Additionally, as regards performance and detection bias, the risk of bias is unclear (Higgins et al., 2011). Concerning performance bias, it is important that neither the researchers nor the participants are aware of the condition to which the participant belongs during the research process, however, since most of the research teams are also responsible for the intervention delivery it is not possible to guarantee full blindness (56.41%), thus increasing the risk of bias. The same constraints were present for detection bias, leading to a silence within most studies regarding the procedures used to ensure blindness of outcome assessment (51.28%). Nevertheless, among those that stated some information regarding detection bias, most ensured blindness of outcome assessment (33.33%), revealing a low risk of bias. Moreover, with regards to control for attrition bias, most of the studies did not specify this information (53.85%) leading to unclear risk, but the majority of those reporting this bias explained the reasons for the attrition and also discussed how missing data had been handled (43.59%). Lastly, only one study did not report full data (i.e., Benn et al., 2012), leading to a low risk of reporting bias within the pool of the 39 interventions considered.

Since one of the eligibility criteria required quasi-experimental or experimental designs, all the studies considered presented at least pre-posttest data. Additionally, 10 studies also presented follow-up assessments (25.64%), ranging from 4 weeks to 1 year after posttest. As for the typology of measures used to assess the impact of the interventions on teachers' outcomes, 79.49% of the studies resorted to self-report measures, 35.90% used external observation measures, 17.95% presented physiological indicators and 7.69% applied behavioral tasks for assessment. In the 39 interventions reviewed, 12 used combined measures to test impacts on the variables assessed (30.77%).

Lastly, as regards outcomes, most of the studies evaluated the impact of the interventions on teachers' well-being (61.54%) and psychological distress (58.97%). In opposition, physical distress was the less gauged domain (28.21%). Furthermore, 26 out of the 39 interventions measured variables from more than one domain, while the impact of the remaining 13 interventions was tested on indicators from only one domain, namely classroom climate and instructional practices (92.31%) and psychological distress (7.69%).

### Meta-Analysis Results

**Figure 1:**
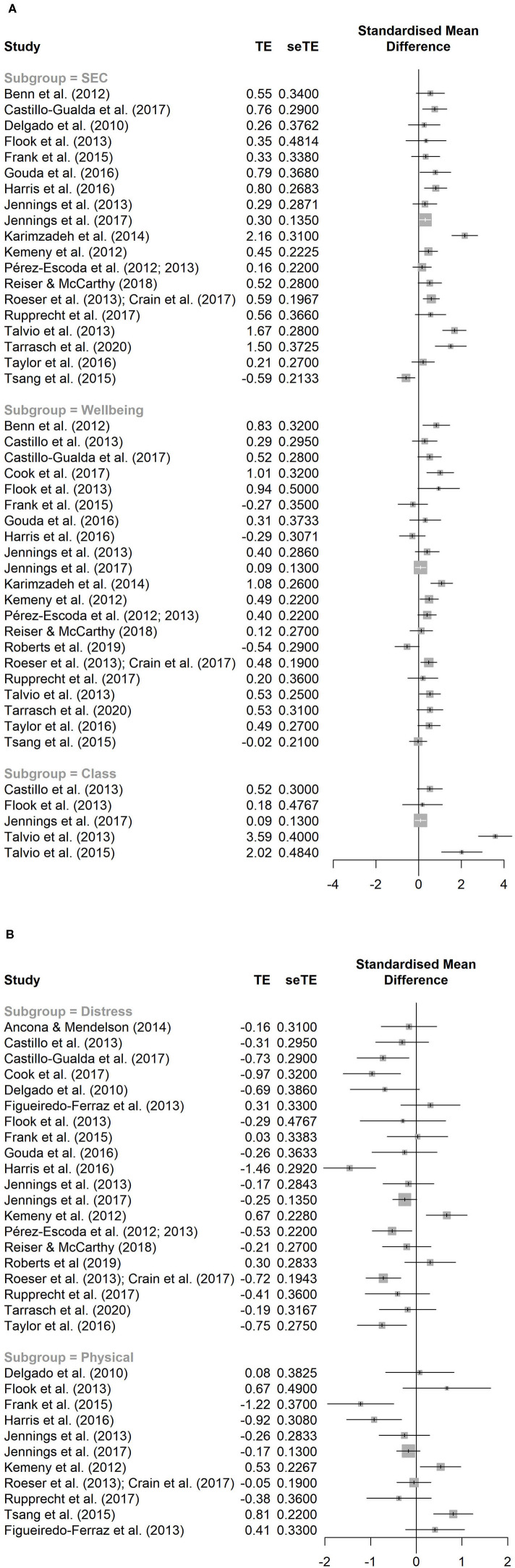
**(A)** Forest plot with weighted average effects for the SEC, Well-being, and Classroom climate and instructional practices domains by study of interventions only targeting teachers. **(B)** Forest plot with weighted average effects for the Psychological distress and Physical distress domains by study of interventions only targeting teachers.

Firstly, the subsample of the 25 SEL interventions (across 27 studies) which only targeted teachers was considered for the meta-analysis procedure. Among the 25 pooled studies included in the meta-analysis, 249 effects were estimated at time 2, revealing a high level of within-study interdependence, which was taken into account using RVE. With regards to H1, significant effects were found for SEC, Psychological distress and Well-being, with Physical distress and Classroom climate and instructional practices being the only figures without statistical significance. Moderate heterogeneity was found for Psychological distress. All other effects revealed high heterogeneity, particularly Classroom climate and instructional practices. Prediction intervals were also wide. No significant effects were found for studies reporting follow-up measures (see Table 4). Figures 1A,B depict forest plots with averaged effects for each domain by plotted study.

**Table 4:** Weighted average effects with heterogeneity estimates and prediction intervals.

	**Individual**				**Combined**			
	***g* (SE)**	**95% CI**	***I^**2**^***	**Prediction Interval**	***g* (SE)**	**95% CI**	***I^**2**^***	**Prediction Interval**
**T2**								
SEC (*n* = 19, *k* = 79 / *n* = 2, *k* = 14)	0.59 (0.14)	[0.29, 0.90]	85.80%	[−0.83, 2.02]	0.04 (0.11)	[−1.40, 1.48]	59.20%	[−5.65, 5.73]
Psychological distress (*n* = 20, *k* = 69 / *n* = 2, *k* = 3)	−0.34 (0.11)	[−0.57, −0.10]	75.50%	[−1.37, 0.69]	−0.02 (0.34)	[−4.35, 4.31]	59.40%	[−6.98, 6.94]
Physical distress (*n* = 11, *k* = 37 / *n* = 0, *k* = 0)	−0.04 (0.19)	[−0.47, 0.38]	88.10%	[−1.68, 1.06]				
Well-being (*n* = 21, *k* = 49 / *n* = 3, *k* = 7)	0.35 (0.09)	[0.16, 0.54]	77.30%	[−0.67, 1.37]	0.34 (0.27)	[−0.92, 1.59]	83.90%	[−2.01, 2.69]
Classroom climate and instructional practices (*n* = 5, *k* = 15 / *n* = 12, *k* = 71)	1.26 (0.68)	[−0.61, 3.14]	96.00%	[−3.59, 6.11]	1.20 (0.61)	[−0.14, 2.53]	98.20%	[−3.49, 5.89]
**Follow-up**								
SEC (*n* = 4, *k* = 11/ *n* = 0, *k* = 0)	0.17 (0.25)	[−0.63, 0.98]	80.00%	[−1.49, 1.83]				
Psychological distress (*n* = 2, *k* = 10/ *n* = 1, *k* = 1)	−0.71 (0.18)	[−3.05, 1.63]	62.50%	[−6.58, 5.16]				
Physical distress (*n* = 2, *k* = 10/ *n* = 0, *k* = 0)	−0.12 (0.17)	[−2.28, 2.05]	47.40%	[−4.31, 4.08]				
Well-being (*n* = 5, *k* = 12/ *n* = 1, *k* = 3)	0.49 (0.26)	[−0.24, 1.22]	78.90%	[−1.10, 2.07]				
Classroom climate and instructional practices (*n* = 0, *k* = 0/ *n* = 4, *k* = 20)					1.26 (0.73)	[−1.07, 3.59]	96.30%	[−3.55, 6.07]

*n (number of studies); k (number of within effects) with values to the left of the bar referring to individual interventions, and values to the right of the bar referring to combined interventions*.

**Figure 2:**
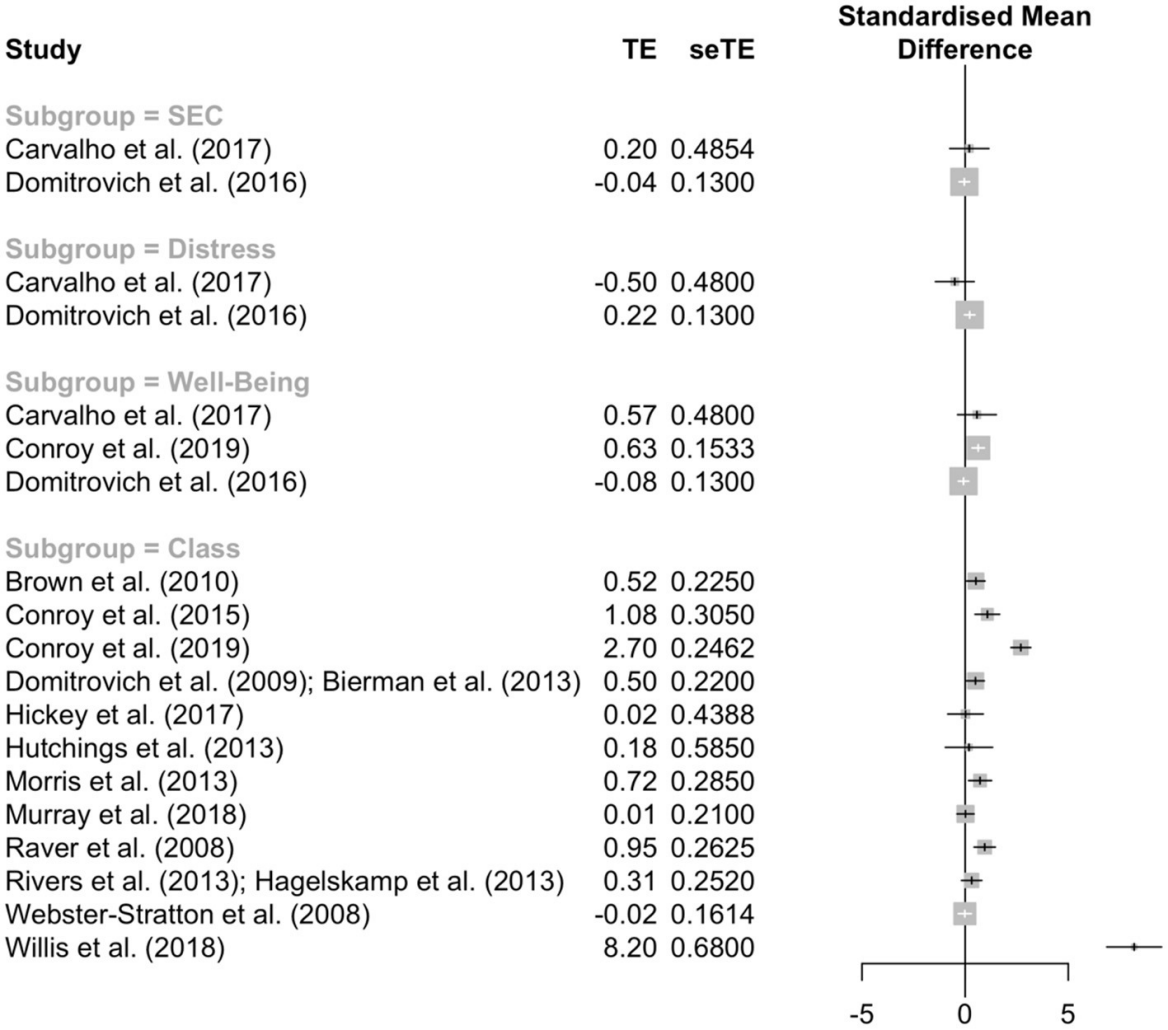
Forest plot with weighted average effects of the five accessed domains by study of combined interventions.

An additional meta-analysis was performed for studies offering a combined intervention. A pool of 14 studies comprising 95 effects revealed non-significant effects (see Table 4). Figure 2 depicts the forest plot with average effects by plotted study.

Meta-regression estimates to explore the role of covariates are shown in Table 5 for studies targeting teachers at time 2. Due to the smaller number of studies and sample requirements (minimum of 10 studies) for simultaneously testing explanatory variables (Thompson and Higgins, 2002), the models were only adjusted for the dimensions with higher number of studies (i.e., SEC, Psychological distress and Well-being). In relation to H2, no significant effects were found regarding the role of covariates.

**Table 5:** Meta-regression models for covariates.

	**Individual**					
	**SEC**		**Psychological distress**		**Well-being**	
	***B(SE)***	**95% CI**	***B(SE)***	**95% CI**	***B(SE)***	**95% CI**
Intercept	0.13(0.66)	[−2.16, 2.42]	−0.07(0.30)	[−0.80, 0.66]	0.50(0.40)	[−0.50, 1.50]
Dosage (15–29 h)	1.07 (0.69)	[−2.24, 4.39]	−0.36 (0.27)	[−1.01, 0.30]	−0.06 (0.42)	[−1.22, 1.10]
Dosage (≥30 h)	0.86 (0.75)	[−1.97, 3.68]	−0.25 (0.29)	[−0.97, 0.47]	−0.16 (0.41)	[−1.24, 0.91]
Cross-training (yes)	0.10 (0.23)	[−0.42, 0.63]	0.42 (0.33)	[−0.34, 1.18]	−0.16 (0.28)	[−0.78, 0.46]
Teaching grade (class)	−0.23 (0.39)	[−1.20, 0.75]	0.01 (0.26)	[−0.62, 0.64]	−0.10 (0.25)	[−0.70, 0.49]
Teaching grade (discipline)	0.40 (0.41)	[−0.60, 1.39]	−0.33 (0.30)	[−1.03, 0.38]	−0.34 (0.37)	[−1.19, 0.51]
Mindfulness (yes)	−0.64 (0.42)	[−1.65, 0.36]	−0.18 (0.27)	[−0.86, 0.51]	0.24 (0.29)	[−0.42, 0.90]

*For dosage, the reference group is 1–14 h; for cross-training, the reference group is no; for grade, the reference group is both; for use of mindfulness techniques, the reference group is no*.

Finally, to assess publication bias, the Vevea and Woods (2005) sensitivity analysis was performed. For interventions only targeting teachers, there was a pattern of publication bias for SEC and Well-being effects. Both effects when corrected for publication bias decreased to 0.44 and 0.24, respectively. As for Psychological distress, the effect remained unaltered (−0.34) after the model correction.

## Discussion

Nowadays, teachers are faced with an imbalance of teaching demands (e.g., workload, classroom management, and interpersonal conflicts) and resources (e.g., teacher training), which impacts their personal lives and job performance (Jennings and Greenberg, 2009). Additionally, teaching-specific stressors have been referred to as mainly socio-emotional related (Roeser et al., 2013), thus leading to researchers worldwide investing in the development of SEL interventions to promote teachers' SEC (Schonert-Reichl, 2017). Nevertheless, to our knowledge, no studies had yet overviewed the impacts of SEL interventions for teachers on their personal and/or professional outcomes. Therefore, it was the aim of this research to review the existing evidence on the effects of SEL interventions on teachers' outcomes. To this end, following an in-depth literature research, a systematic review with meta-analysis was performed on 43 empirical studies (with a total of 39 interventions).

In order to achieve our goal, two research questions and two hypotheses were established. With regards to Q1, in keeping with other findings for SEL interventions for students (Tolan et al., 2016), the pooled studies did not, in their majority, clearly state the theoretical foundations to which they resorted to inform the design and implementation of their SEL interventions for teachers. Only approximately one third of the eligible studies presented clear information on the theoretical frameworks which guided the development of the SEL intervention used. This is particularly important, since it can nurture heterogeneity and blur the frontiers of the SEL rationale, making it more difficult to compare the interventions with each other, thus limiting the estimate of robust and secure effects. Furthermore, this concern extends to the intervention and research procedures adopted which may contribute to improving the empirical-evidence of SEL intervention effects, namely by controlling for biases (Biglan et al., 2003; Higgins et al., 2011). Even though most of the studies presented some type of randomization, suggesting a low risk of selection bias (Higgins et al., 2011), with regards to the remaining procedures, the level of bias is more unclear. Most of the studies were not led by independent research teams and did not report fidelity assessment. Moreover, since the pooled studies resorted to the assessment of interventions' effects and most interventions were delivered by the researchers themselves, consequently most of the studies did not ensure full blindness of the participants and outcomes, which may increase the risk of bias (Higgins et al., 2011). Additionally, most of the studies did not present data from follow-up assessments and the data collection was mostly conducted through self-report instruments. Therefore, regarding Q2, it may be concluded that there is a need for future studies to improve their quality in terms of methodological processes which may ensure higher quality and validity of the SEL interventions' contributions.

With regards to the first hypothesis of the study, the results revealed that H1 was partially sustained. The findings indicated statistically significant medium effects of SEL interventions for teachers on SEC (*g* = 0.59), Well-being (*g* = 0.35), and Psychological distress (*g* = −0.34). These results are in line with the prior research that highlights the contribution of SEL interventions for teachers to teachers' personal and job performance-related dimensions (Schonert-Reichl, 2017). Impacts on Physical distress and Classroom climate and instructional practices were found to be non-significant. The absence of significant effects in the Physical distress dimension at posttest may be due to the fact that changes at the behavioral and physiological indicators' level emerged following a prior psychological change (e.g., perception). Thus, the aforementioned changes may take longer to appear (Tsang et al., 2015). On the other hand, the non-significant effects on Classroom climate and instructional practices may be associated with the high heterogeneity observed for this domain. Since the vast majority of studies assessing this domain used a multilevel approach to control for possible context effects (e.g., school in which the teachers were integrated) and were mainly homogeneous in terms of the specific strategies used in each intervention and their target, an alternative explanation for the extreme variance found between studies may be related to the approach used for the data collection. In fact, the studies evaluating the Classroom climate and instructional practices domain did so mainly through observation measures, without triangulating this data with data from other sources (e.g., informant-report measures) and other domains (e.g., SEC), which may have contributed to an increased bias and affected the results, causing more heterogeneity of effects. Future research should, therefore, be cautious when assessing SEL interventions' impact on teaching practices, namely ensuring that observations of teachers' behavior within their classroom are made by independent observers, multiple sources of data are gathered, and data from multiple domains (e.g., teachers' SEC) are crossed.

Moreover, there were no significant effects at follow-up. However, the pool of studies eligible for the assessment of stability and sleeper effects was small and, therefore, conclusions should be drawn with caution.

Additionally, combined interventions did not present significant impacts on teacher-level outcomes. This result may derive from the origin of this type of intervention. Combined SEL interventions are mainly developed targeting the students' SEC. Nevertheless, recognizing the role of teachers in students' development, some interventions also integrate an intervention (usually prior to the students' intervention) targeting teachers to help them react more socially and emotionally in their classrooms. Consequently, these modules targeting teachers' SEC are usually shorter and place greater emphasis on inter-personal SEC (i.e., social awareness and relationship skills) which may affect the results.

Furthermore, in accordance with the findings regarding SEL interventions targeting students, where significant effects where mainly found in children's SEC (*g* = 0.57; Durlak et al., 2011), our results showed that SEL interventions for teachers also had a higher impact on their SEC (*g* = 0.59), and a similar effect. Additionally, these findings suggest that SEL interventions for teachers also serve to improve perceived personal well-being and positive emotions and to reduce perceived psychological discomfort and internalizing problems, sustaining preventive action for ill-health issues (e.g., burnout) and promotive action for well-being and mental health (e.g., personal accomplishment, job satisfaction).

The findings suggest that this intervention approach may contribute to improving targeted outcomes for preK-12 in-service teachers. Nevertheless, the heterogeneity among the studies was high across the domains (*I*^2^ > 75.00%), suggesting the existence of covariates. In line with the aforementioned, prior literature has also found high levels of heterogeneity among SEL interventions for children (*I*^2^ = 91%; Durlak et al., 2011).

Lastly, in order to address H2, we tested for the impact of covariates. This hypothesis was rejected. Even though high heterogeneity levels among the studies were found, suggesting that more than 75% of between-study variance may be attributed to predictor variables, the tested potential covariates did not significantly impact treatment efficacy. This finding may be due to the covariates that were selected (despite the selection being theoretically grounded), and to the methodological and conceptual issues that emerged across the studies (e.g., psychometric properties of the selected instruments, sample size that may compromise the power of the analysis computed). Also, due to the smaller number of studies and sample requirements, the test of the covariates' prediction effect presented limitations and could not be performed across all the domains. Consequently, more research is required to understand precisely which variables may be explaining the found variance. Furthermore, more in-depth research is needed in order to test and expand knowledge regarding the particular role of the considered covariates in SEL interventions for in-service teachers' impact on teachers' outcomes.

Furthermore, this study highlights some important methodological and conceptual aspects that should be addressed in future research in this area. First, our findings reinforce the current lack of research on SEL interventions for teachers' efficacy and the need for greater homogeneity of practices (Jennings et al., 2017). Additionally, our results revealed that most of the studies relied on small samples (*N* < 100), compromising the power of the computed analyses. Moreover, the majority of the eligible papers considered only self-report measures, which are affected by random reporting, social desirability, and may also mask the impact on SEC, as previously stated. Therefore, future research should test effects on larger samples and through multiple data collection methods (e.g., behavioral measures, informant-report measures, and direct observations). Furthermore, even though we only considered empirical studies, only two (i.e., Domitrovich et al., 2016; Roberts et al., 2019) used an active control group (i.e., alternative intervention) in addition to a passive control group (e.g., waiting list); few studies included follow-up assessments; and a minority of studies (<20%) presented information on fidelity in the delivery of the intervention. These methodological shortcomings are particularly problematic since they may compromise control of the Hawthorne effect, the study of maintenance and sleeper effects, as well as the identification of evidence-based practices and determinant components, which influence outcomes. Lastly, with regard to conceptual features, few of the studies explicitly presented the characteristics and contents of the interventions and the strategies used, and all the interventions were designed at an individual / micro level (i.e., targeting only teachers or teachers and their students), and these features may have had an important effect on the results found. Although these findings provided initial orientations as regards the factors taken into consideration during SEL interventions for teachers' development, in order to achieve the aspired results, further research is needed to deepen, validate and reinforce our results.

### Limitations and Future Research

This study presents some limitations that should be considered when interpreting the data. First, due to the eligibility criteria, some research studies were excluded since they did not provide enough data for the estimation of effect sizes. Likewise, studies with non-experimental designs which used combined samples (i.e., teachers and other working professionals), papers that had not been published in peer-reviewed journals and gray literature were excluded. Although these options contributed to ensuring the quality of the research, they also may have led to a bias of the findings (Higgins et al., 2019). Resorting specifically to gray literature, its inclusion alone may, paradoxically, introduce bias (Higgins et al., 2019). Therefore, since the SEL rationale, and the interventions developed within this approach, are already heterogeneous and need further integration, the option was taken to minimize the possible entropy through the restriction of eligible studies, in order to guarantee the validity of the meta-analytic procedure. However, considering the expanding research in this area, it is important for future research to conduct replications of this initial meta-analysis in order to extend our findings. Moreover, due to the heterogeneity of contents addressed by the interventions and outcomes assessed, we were not able to test for finer effects. It would be interesting for future research studies to analyze specific effects on the sub-dimensions of outcome domains (e.g., within the scope of psychological distress, understanding SEL interventions' impact on teachers' burnout levels), and to test for possible distinct effects among different SEC-related areas (e.g., self-regulation). Thus, future meta-analyses should investigate the specific pathways our analysis did not take, due to its wider view, with greater precision.

As for the analysis itself, high levels of heterogeneity among the studies were found, suggesting that between-study variance was explained by covariates. However, despite the fact that the predictors' selection was based on prior literature recommendations, the tested covariates did not contribute to explaining the heterogeneity found. Moreover, the 95% range of prediction intervals contain values below and above 0, meaning effects in new studies may be on the opposite side of the summary point estimate presented in the current meta-analysis. This is consistent with the high heterogeneity found, which tends to be higher for continuous outcomes (IntHout et al., 2016). Despite the high heterogeneity of the effects, they do not appear to be explained by the covariates deemed relevant in the literature, thus suggesting the need for future research to explore other predictors. This result also points to the need to develop far more theoretically adjusted interventions, since a great diversity of forms of implementation, theoretical frameworks, and methodological procedures (e.g., data collection protocols used, outcomes assessed, fidelity assessment, and control for risk of biases) regarding SEL interventions' implementation and evaluation was observed. Hence, in this context, this systematic review with meta-analysis contributed particularly to inform and highlight the need to build more solid and well theoretically grounded SEL interventions for teachers.

Moreover, there are several statistical methods to evaluate publication bias, as may be observed in the work of Renkewitz and Keiner (2019), which shows the non-existence of a single best detection method, and that no detection method yields “proof” of bias. We used Vevea and Woods (2005) approach as a sensitivity analysis, and a decrease was found in the point estimates for the SEC and Well-being domains, suggesting a pattern of publication bias. This finding reinforces the aforementioned cautiousness of the achieved results and the need to further promote the theoretical and methodological soundness of interventions (Renkewitz and Keiner, 2019). Additionally, this result (along with the absence of publication bias for the Psychological distress domain, which also presents the lowest levels of heterogeneity) stresses the importance of a careful and more robust selection of methodological procedures. More specifically, the suggestion of more consistency of the data within the Psychological distress domain may be due to the fact that, there is, in fact, less divergence of the variables assessed in this domain, and also a tendency to use the same well-established instruments (e.g., the Maslach Burnout Inventory to evaluate burnout symptoms).

### Theoretical and Practical Implications

Bearing the aforementioned restrictions in mind, SEL interventions for teachers appear to have, on average, moderate impacts on improving teachers' SEC and Well-being, and reducing their Psychological distress symptoms. Taken together, these results reinforce the potential of SEL for teachers' personal and professional outcomes, thus corroborating the relevance of including SEL approaches in teacher training. Also, due to its favorable contributions for teachers' well-being and job performance, these findings also sustain and reinforce the importance to future studies to review, reflect and discuss the need to explicitly integrate SEL for pre-service and novice teachers initial preparation (Schonert-Reichl et al., 2017).

In addition to illustrating how this type of intervention may play a significant role in teacher training and consequent performance, our findings allow us to draw some insights as regards the design of research studies. Firstly, the results emphasize the importance of developing more theoretically and methodologically robust SEL interventions for teachers, in order to ensure higher quality and validity of the research and provide better and more reliable empirical evidence of SEL intervention effects (Biglan et al., 2003; Higgins et al., 2011). It is, therefore, important for future research on the efficacy of SEL interventions to evaluate maintenance and sleeper effects more consistently, namely through the inclusion of follow-up assessment. Moreover, due to the between-studies variance and suggestion of publication bias for the SEC and Well-being domains, it may be important to reflect on the instruments used to measure the assessed constructs. It may be the case that since the eligible studies tested highly heterogeneous and distinct variables through multiple instruments, the found variance may reflect an inconsistency in the evaluation procedures. Thus, a more suitable match between the selected instruments and the variables under study should be a concern for future studies. Additionally, it is crucial to align the selected variables with the intervention objectives and addressed contents. Most of the studies showed a mismatch among these three methodological aspects or did not provide enough information on the objectives and contents of the interventions, making this relationship unclear. Moreover, research using repeated measures and longitudinal designs is needed to test other potential moderators and mediation effects that help to extend current knowledge and develop more robust guidelines for these types of interventions.

Likewise, our results provide important clues for the development of specific guidelines on the design of SEL interventions for teachers. Firstly, the inconsistent and mostly silent results regarding the theoretical frameworks used to ground the intervention immediately places limitations on the comparison and evaluation of the SEL interventions for teachers. The theoretically based interventions are, nevertheless, considered one of the main features of good practices (e.g., Durlak et al., 2015).

Moreover, the predictive role of the specific content features found in this study appears to indicate that, as regards SEL interventions for teachers, the customization of contents to specific groups of teachers (i.e., class-level and discipline-level teachers) did not play a significant role in the assessed domains. Therefore, tailoring the intervention to a specific group of teachers (i.e., class-level or discipline-level teachers) does not appear to be particularly relevant. However, regardless of the chosen approach, SEL interventions should provide opportunities for teachers to be involved in activities that explicitly promote reflection and perspective taking in a group setting, thus enabling them to share ideas and experiences. Irrespective of these insights, results were not tested across all the domains (due to data conditionings), and therefore, more research is needed to replicate and verify these factors.

Furthermore, results suggest that, contrary to what was expected, dosage, cross-session training [as suggested by Gulamhussein (2013)] and the use of mindfulness techniques [as suggested by Klingbeil and Renshaw (2018)] did not predict the effect of SEL interventions. Although additional research is needed to clarify these potential influences, the findings of this study should be considered by intervention developers when planning the structure and features of new interventions. For example, perhaps a more effective duration (i.e., distance between 1st and last training session) and frequency of the intervention may play a more crucial role in SEL interventions for teachers' effectiveness than a higher number of formal training hours (i.e., dosage). Also, despite the promising results found in prior literature (e.g., Klingbeil and Renshaw, 2018), the use of mindfulness techniques applied to the development of SEC appears to have a similar effect to that of traditional SEL techniques.

Finally, it is also important to note that all the eligible interventions were designed at an individual / micro level, at best including both teachers and students, and using a school-based primary level approach. No interventions designed at an organizational level (i.e., involving all school-community members) were found, or any that considered the baseline level of teachers' SEC. Therefore, since prior literature underlines the importance of promoting changes at an organizational level in order to produce long-lasting improvements (e.g., Durlak et al., 2015), there is a need for future research on SEL interventions for teachers to consider the contribution and effectiveness of organizational-level interventions. Likewise, as interventions to date have been developed on the basis of universal level approaches, it may be important to adjust the interventions to teachers' baseline characteristics and sketch SEL interventions according to a multi-tiered approach. Within this scenario, as more methodologically rigorous studies emerge, research including meta-analytic reviews should be conducted to refine and extend our findings.

## Data Availability Statement

The original contributions presented in the study are included in the article/Supplementary Material, further inquiries can be directed to the corresponding author/s.

## Author Contributions

SO: designed and executed the study, analyzed the data, wrote, edited, and revised the manuscript. MSR: assisted with the design, collaborated with the data analyses, the writing, and the editing of the final manuscript. NSP: collaborated with the data analyses and the writing of the manuscript. AM-P: assisted with the design and execution of the study, collaborated with the data analyses, the writing, and the editing of the final manuscript. AMV-S: assisted with the design and execution of the study, collaborated with the writing, and editing of the final manuscript. All authors approved the final version of the manuscript for submission.

## Conflict of Interest

The authors declare that the research was conducted in the absence of any commercial or financial relationships that could be construed as a potential conflict of interest.
