# Supplementary material for: Impacts of Social and Emotional Learning Interventions for Teachers on Teachers' Outcomes: A Systematic Review With Meta-Analysis
Source: Front Psychol. 2021 Jul 1;12:677217. doi: 10.3389/fpsyg.2021.677217 (PMC8281129; doi:10.3389/fpsyg.2021.677217)

Supplementary Online Material

Expanded methodological procedures, Supplementary results, and References for the reviewed interventions

[To accompany Oliveira, S., Roberto, M. S., Pereira, N., Marques-Pinto, A., & Veiga-Simão, A. M. (2021). Impacts of social and emotional learning interventions for teachers on teachers' outcomes: A systematic review with meta-analysis. *Frontiers in Psychology*. https://doi.org/10.3389/fpsyg.2021.677217]

The following supplementary online material includes: greater detail on the methodological procedures adopted in the study (i.e., information on accessed databases, selected descriptors, initial search results and data collection process, eligibility criteria, and variables included in the coding process concerning both quality in controlling bias of the selected records and specific characteristics / results); the complete reference list regarding the final data set of selected studies; the initial results (i.e., a synthesis of the information collected from the final data set of selected studies), and the funnel plots which help inform on possible publication bias.

1 Expanded Methodological Procedures

1.1 Accessed Databases

1.1.1 EBSCOhost web which provides access to the following research databases for Academic Libraries on Psychology and Education: APA PsycArticles; APA PsycBooks; APA PsycExtra; APA PsycInfo; APA PsycTests; APA PsycTherapy; British Education Index; Education Abstracts; Education Full Text; Education Index Retrospective: 1929-1983 (Archive); Education Research Complete; Education Source; Educational Administration Abstracts; ERIC; Health and Psychosocial Instruments (HaPI); Mental Measurements Yearbook; Mental Measurements Yearbook with Tests in Print; Mental Measurements Yearbook with Tests in Print Internacional; PEP Archive; PSICODOC; Psychology & Behavioral Sciences Collection; PSYNDEX: Literature and Tests; Research Starters – Education (for further information: https://www.ebsco.com/products/research-databases?search=&f%5B0%5D=market%3A1&f%5B1%5D=subject%3A6351&f%5B2%5D=subject%3A6411&page=0)

1.1.2 b-ON which provides access to the following research databases for scientific and technological research institutions and higher education institutions: Academic Search Complete; American Chemical Society; American Institute of Physics; Annual Reviews; Association for Computing Machinery; Association for Computing Machinery; Business Source Complete; Coimbra University Press; Current Contents (ISI); Elsevier; Essencial Science Indicators (ISI); ERIC; IEEE; Institute of Physics; Journal Citation Reports (ISI); LISTA; Nature; Royal Society of Chemistry; Sage; Society for Industrial and Applied Mathematics; Springer; Taylor & Francis; Web of Science; Wiley; Zentralblatt (for further information: https://www.b-on.pt/colecoes/#tabela1)

1.1.3 **SCOPUS** which is the largest abstract and citation peer-reviewed literature citation and indexing database, including scientific journals, books, conferences, with around 70 million indexed documents (for further information: https://service.elsevier.com/app/answers/detail/a_id/15534/supporthub/scopus/#tips).

1.1.4 **SciELO** which is a cooperative decentralized database addressing the electronic publication of scientific journals, with an emphasis on those produced by Ibero-American countries (for further information: https://scielo.org/en)

These platforms and databases were selected in order to enable both the representation of global publications (i.e., from the northern and southern hemispheres), and the coverage of the most representative databases for Psychology and Education publications [e.g., PsycARTICLES, PsycINFO, ERIC, Web of Science, IEEE, SciELO, Scopus and ScienceDirect (provided through Elsevier)].

1.2 Detailed Eligibility Criteria

Records were considered eligible for this study whenever:

1. Recourse to a SEL intervention targeting in-service preK-12 teachers was found.
   Studies targeting university and / or pre-service teachers, and studies which, when using combined samples (e.g., in-service teachers and school staff) did not provide data regarding only the teachers group, were excluded.

2. The intervention was developed in accordance with the rationale and operationalization underlying this study.
   The record had to specifically indicate that the program / intervention had been developed in accordance with the SEL rationale as originally proposed by Elias and colleagues (1997) and further presented in Durlak and colleagues (2015). Otherwise, to ensure homogeneity within the research analyzed, records were not considered eligible.

3. The intervention aimed to directly enhance at least one of the five considered SEC in in-service teachers.
   The program / intervention under review had to specifically state the enhancement of in-service teachers' own SEC as an aim or strategy, in one or more of the five key-competencies considered in this study (i.e., self-awareness, self-management, social awareness, relationship skills, and responsible decision making). Nevertheless, the report was not required to include a measure of SEC in its outcomes. All the interventions aiming solely to train teachers to promote SEC in their students (e.g., teaching teachers how to apply a program to develop students' SEC) were not deemed eligible, even though findings of the indirect effects on teachers' SEC development by means of these interventions may be expected.

4. The efficacy of the SEL intervention was assessed through teacher-level variables, regarding personal and / or occupational outcomes.
   Studies which did not access impacts on teacher-level variables were not included.

5. The efficacy of the intervention was assessed through a quasi-experimental or experimental design.
   To minimize the possible bias associated with underestimation or overestimation of the true intervention effect, studies were not included if at least two comparison groups (i.e., control vs treatment) and two points of assessment (i.e., pre and post-test) were not ensured.

6. The report contained sufficient information to calculate the effect sizes of the interventions' impacts.
   In order to be eligible to integrate the meta-analytic procedure, the report had to present enough information to estimate the effect size for at least one outcome.

7. The full-text of the report was available and published in Psychology or Educational peer-review journals.

To minimize the possible bias associated with underestimation or overestimation of the true intervention effect, studies had to be published in peer-review journals to be considered eligible. Thus, grey literature and studies published in non-peer-review journals were not eligible for the present study. This option was made in order to minimize the possible entropy caused by heterogeneity of data (Higgins et al., 2019), which is already expected since the SEL rationale itself requires further integration in order to guarantee the validity of the meta-analytic procedure.

8. The report was published after 1995.
   Since the terms "SEL" and "emotional intelligence" were only developed and presented to the public in 1995 (with the publication of Goleman's book *Emotional Intelligence*), the timespan was narrowed in order to only include programs which may have been developed in accordance with the rationale underlying the present study.

2 Initial search results

Table S1

Search results by databases and descriptors

**Descriptors combination used:**<br><br>**a) for EBSCO and b-ON:** TI (teacher OR teachers OR educator OR educators OR professor OR professors) AND SU (social and emotional learning OR social and emotional development OR social and emotional education OR social and emotional training OR social and emotional skills OR social and emotional competence OR socioemotional learning OR socioemotional development OR socioemotional education OR socioemotional training OR socioemotional skills OR socioemotional competence OR socio-emotional learning OR socio-emotional development OR socio-emotional education OR socio-emotional training OR socio-emotional skills OR socio-emotional competence OR social learning OR social development OR social education OR social training OR social skills OR social competence OR emotional learning OR emotional development OR emotional education OR emotional training OR emotional skills OR emotional competence OR SEL OR SEL development OR SEL education OR SEE OR SEL training OR SEL skills OR SEL competence) AND SU (effective program OR effective intervention OR effective treatment OR program efficacy OR intervention efficacy OR treatment efficacy OR program effectiveness OR intervention effectiveness OR treatment effectiveness OR program evaluation OR intervention evaluation OR treatment evaluation) NOT TI (student OR students OR child OR children OR adolescent OR adolescents)<br><br>**b) for SCOPUS:** (TITLE ((teacher) OR (teachers) OR (educator) OR (educators) OR (professor) OR (professors)) AND TITLE-ABS-KEY ((social and emotional learning) OR (social and emotional development) OR (social and emotional education) OR (social and emotional training) OR (social and emotional skills) OR (social and emotional competence) OR (socioemotional learning) OR (socioemotional development) OR (socioemotional education) OR (socioemotional training) OR (socioemotional skills) OR (socioemotional competence) OR (socio-emotional learning) OR (socio-emotional development) OR (socio-emotional education) OR (socio-emotional training) OR (socio-emotional skills) OR (socio-emotional competence) OR (social learning) OR (social development) OR (social education) OR (social training) OR (social skills) OR (social competence) OR (emotional learning) OR (emotional development) OR (emotional education) OR (emotional training) OR (emotional skills) OR (emotional competence) OR (SEL) OR (SEL development) OR (SEL education) OR (SEE) OR (SEL training) OR (SEL skills) OR (SEL competence)) AND TITLE-ABS-KEY ((effective AND program) OR (effective AND intervention) OR (effective AND treatment) OR (program AND efficacy) OR (intervention AND efficacy) OR (treatment AND efficacy)) OR TITLE-ABS-KEY ((program AND effectiveness) OR (intervention AND effectiveness) OR (treatment AND effectiveness) OR (program AND evaluation) OR (intervention AND evaluation) OR (treatment AND evaluation)) AND NOT TITLE ((student) OR (students) OR (child) OR (children) OR (adolescent) OR (adolescents))) AND DOCTYPE (ar OR re) AND PUBYEAR > 1994 AND (LIMIT-TO (SUBJAREA , "PSYC") OR LIMIT-TO (SUBJAREA , "SOCI")) AND (LIMIT-TO (DOCTYPE , "ar"))<br><br>**c) for SciELO:** (ti:((teacher) OR (teachers) OR (educator) OR (educators) OR (professor) OR (professors))) AND (ab:((social and emotional learning) OR (social and emotional

development) OR (social and emotional education) OR (social and emotional training) OR (social and emotional skills) OR (social and emotional competence) OR (socioemotional learning) OR (socioemotional development) OR (socioemotional education) OR (socioemotional training) OR (socioemotional skills) OR (socioemotional competence) OR (socio-emotional learning) OR (socio-emotional development) OR (socio-emotional education) OR (socio-emotional training) OR (socio-emotional skills) OR (socio-emotional competence) OR (social learning) OR (social development) OR (social education) OR (social training) OR (social skills) OR (social competence) OR (emotional learning) OR (emotional development) OR (emotional education) OR (emotional training) OR (emotional skills) OR (emotional competence) OR (SEL) OR (SEL development) OR (SEL education) OR (SEE) OR (SEL training) OR (SEL skills) OR (SEL competence))) AND (ab:((effective program) OR (effective intervention) OR (effective treatment) OR (program efficacy) OR (intervention efficacy) OR (treatment efficacy) OR (program effectiveness) OR (intervention effectiveness) OR (treatment effectiveness) OR (program evaluation) OR (intervention evaluation) OR (treatment evaluation))) AND NOT (ti:((student) OR (students) OR (child) OR (children) OR (adolescent) OR (adolescents))) AND wok_subject_categories:("education & educational research" OR "psychology, multidisciplinary" OR "education, scientific disciplines" OR "education, special" OR "psychology" OR "psychology, applied" OR "psychology, clinical")

**Database**	**Number of hits**
EBSCOhost web	64
b-ON	33
SCOPUS	619
SciELO	18
Other (i.e., consortia guides, hand search on references list, and organization websites)	40
**Total:**	774

Figure S1
PRISMA diagram displaying data collection process

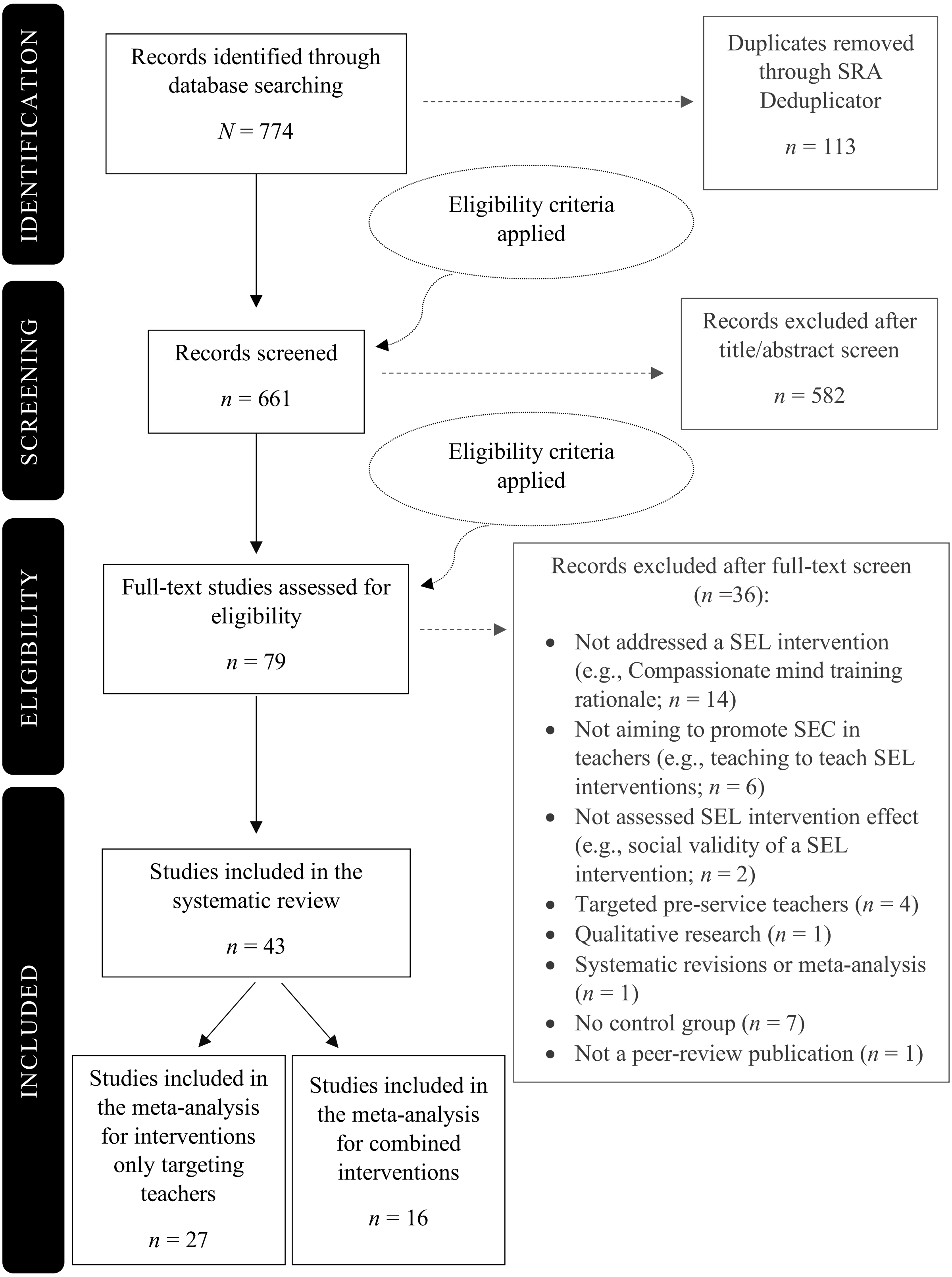

Table S2
References list containing the 43 studies regarding the final data set of the present study

Code	Reference
S01*	Ancona, M. R., & Mendelson, T. (2014). Feasibility and preliminary outcomes of a yoga and mindfulness intervention for school teachers. *Advances in School Mental Health Promotion, 7*(3), 156-170. https://doi.org/10.1080/1754730X.2014.920135
S04	Carvalho, J. S., Marques Pinto, A., & Marôco, J. (2017). Results of a mindfulness-based social-emotional learning program on portuguese elementary students and teachers: A quasi-experimental study. *Mindfulness, 8*(2), 337–350. https://doi.org/10.1007/s12671-016-0603-z
S02*	Benn, R., Akiva, T., Arel, S., & Roeser, R. W. (2012). Mindfulness training effects for parents and educators of children with special needs. *Developmental psychology, 48*(5), 1476-1487. https://doi.org/10.1037/a0027537
S11	Bierman, K. L., Sanford DeRousie, R. M., Heinrichs, B., Domitrovich, C. E., Greenberg, M. T., & Gill, S. (2013). Sustaining high-quality teaching and evidence-based curricula: Follow-up assessment of teachers in the REDI project. *Early Education & Development, 24*(8), 1194-1213. https://doi.org/10.1080/10409289.2013.755457
S03	Brown, J. L., Jones, S. M., LaRusso, M. D., & Aber, J. L. (2010). Improving classroom quality: Teacher influences and experimental impacts of the 4rs program. *Journal of educational psychology, 102*(1), 153-167. https://doi.org/10.1037/a0018160
S05*	Castillo, R., Fernández-Berrocal, P., & Brackett, M. A. (2013). Enhancing teacher effectiveness in Spain: A pilot study of the RULER approach to social and emotional learning. *Journal of Education and Training Studies, 1*(2), 263-272. https://doi.org/10.11114/jets.v1i2.203
S06*	Castillo-Gualda, R., García, V., Pena, M., Galán, A., & Brackett, M. A. (2017). Resultados preliminares del método RULER en la inteligencia emocional y el compromiso laboral de profesores Españoles. *Electronic Journal of Research in Education Psychology, 15*(43), 641-664. https://doi.org/10.14204/ejrep.43.17068
S08	Conroy, M. A., Sutherland, K. S., Algina, J., Ladwig, C., Werch, B., Martinez, J., ... Gyure, M. (2019). Outcomes of the BEST in CLASS intervention on teachers' use of effective practices, self-efficacy, and classroom quality. *School Psychology Review, 48*(1), 31-45. https://doi.org/10.17105/SPR-2018-0003.V48-1
S07	Conroy, M. A., Sutherland, K. S., Algina, J. J., Wilson, R. E., Martinez, J. R., & Whalon, K. J. (2015). Measuring teacher implementation of the BEST in CLASS intervention program and corollary child outcomes. *Journal of Emotional and Behavioral Disorders, 23*(3), 144-155. https://doi.org/10.1177/1063426614532949
S09*	Cook, C. R., Miller, F. G., Fiat, A., Renshaw, T., Frye, M., Joseph, G., & Decano, P. (2017). Promoting secondary teachers' well-being and intentions to implement evidence-based practices: Randomized evaluation of the achiever resilience curriculum. *Psychology in the Schools, 54*(1), 13-28. https://doi.org/10.1002/pits.21980
S31*	Crain, T. L., Schonert-Reichl, K. A., & Roeser, R. W. (2017). Cultivating teacher mindfulness: Effects of a randomized controlled trial on work, home, and sleep outcomes. *Journal of Occupational Health Psychology, 22*(2), 138-152. https://doi.org/10.1037/ocp0000043
S10*	Delgado, L. C., Guerra, P., Perakakis, P., Viedma, M. I., Robles, H., & Vila, J. (2010). Eficacia de un programa de entrenamiento en conciencia plena (mindfulness) y valores humanos como herramienta de regulación emocional y prevención del estrés para profesores. *Behavioral Psychology/Psicologia Conductual, 18*(3), 511-533. Retrieved from https://www.researchgate.net/publication/286036889

S12	Domitrovich, C. E., Bradshaw, C. P., Berg, J. K., Pas, E. T., Becker, K. D., Musci, R., ... Ialongo, N. (2016). How do school-based prevention programs impact teachers? Findings from a randomized trial of an integrated classroom management and social-emotional program. *Prevention Science, 17*(3), 325-337. https://doi.org/10.1007/s11121-015-0618-z
S11	Domitrovich, C. E., Gest, S. D., Gill, S., Bierman, K. L., Welsh, J. A., & Jones, D. (2009). Fostering high-quality teaching with an enriched curriculum and professional development support: The Head Start REDI program. *American Educational Research Journal, 46*(2), 567-597. https://doi.org/10.3102/0002831208328089
S13*	Figueiredo-Ferraz, H., Gil-Monte, P. R., do Couto, B. R., & Grau-Alberola, E. (2013). Influence of a cognitive behavioural training program on health: A study among primary teachers. *Revista de Psicodidáctica, 18*(2), 343-356. https://doi.org/10.1387/RevPsicodidact.6995
S14*	Flook, L., Goldberg, S. B., Pinger, L., Bonus, K., & Davidson, R. J. (2013). Mindfulness for teachers: A pilot study to assess effects on stress, burnout, and teaching efficacy. *Mind, Brain, and Education, 7*(3), 182-195. https://doi.org/10.1111/mbe.12026
S15*	Frank, J. L., Reibel, D., Broderick, P., Cantrell, T., & Metz, S. (2015). The effectiveness of mindfulness-based stress reduction on educator stress and well-being: Results from a pilot study. *Mindfulness, 6*(2), 208-216. https://doi.org/10.1007/s12671-013-0246-2
S16*	Gouda, S., Luong, M. T., Schmidt, S., & Bauer, J. (2016). Students and teachers benefit from mindfulness-based stress reduction in a school-embedded pilot study. *Frontiers in Psychology, 7*. https://doi.org/10.3389/fpsyg.2016.00590
S29	Hagelskamp, C., Brackett, M. A., Rivers, S. E., & Salovey, P. (2013). Improving classroom quality with the ruler approach to social and emotional learning: Proximal and distal outcomes. *American Journal of Community Psychology, 51*, 530-543. https://doi.org/10.1007/s10464-013-9570-x
S17*	Harris, A. R., Jennings, P. A., Katz, D. A., Abenavoli, R. M., & Greenberg, M. T. (2016). Promoting stress management and wellbeing in educators: Feasibility and efficacy of a school-based yoga and mindfulness intervention. *Mindfulness, 7*(1), 143-154. https://doi.org/10.1007/s12671-015-0451-2
S18	Hickey, G., McGilloway, S., Hyland, L., Leckey, Y., Kelly, P., Bywater, T., ... O'Neill, D. (2017). Exploring the effects of a universal classroom management training programme on teacher and child behaviour: A group randomised controlled trial and cost analysis. *Journal of Early Childhood Research, 15*(2), 174-194. https://doi.org/10.1177/1476718X15579747
S19	Hutchings, J., Martin-Forbes, P., Daley, D., & Williams, M. E. (2013). A randomized controlled trial of the impact of a teacher classroom management program on the classroom behavior of children with and without behavior problems. *Journal of School Psychology, 51*(5), 571-585. https://doi.org/10.1016/j.jsp.2013.08.001
S21*	Jennings, P. A., Brown, J. L., Frank, J. L., Doyle, S., Oh, Y., Davis, R., Rasheed, D., … Greenberg, M. T. (2017). Impacts of the CARE for teachers program on teachers' social and emotional competence and classroom interactions. *Journal of Educational Psychology, 109*, 1010-1028. https://doi.org/10.1037/edu0000187
S20*	Jennings, P. A., Frank, J. L., Snowberg, K. E., Coccia, M. A., & Greenberg, M. T. (2013). Improving classroom learning environments by Cultivating Awareness and Resilience in Education (CARE): Results of a randomized controlled trial. *School Psychology Quarterly, 28*(4), 374-390. https://doi.org/10.1037/spq0000035
S22*	Karimzadeh, M., Salehi, H., Embi, M. A., Nasiri, M., & Shojaee, M. (2014). Teaching efficacy in the classroom: Skill based training for teachers' empowerment. *English Language Teaching, 7*(8), 106-115. https://doi.org/10.5539/elt.v7n8p106

S23*	Kemeny, M. E., Foltz, C., Cavanagh, J. F., Cullen, M., Giese-Davis, J., Jennings, P., ... Ekman, P. (2012). Contemplative/emotion training reduces negative emotional behavior and promotes prosocial responses. *Emotion, 12*(2), 338-350. https://doi.org/10.1037/a0026118
S24	Morris, P., Millenky, M., Raver, C. C., & Jones, S. M. (2013). Does a preschool social and emotional learning intervention pay off for classroom instruction and children's behavior and academic skills? Evidence from the foundations of learning project. *Early Education & Development, 24*(7), 1020-1042. https://doi.org/10.1080/10409289.2013.825187
S25	Murray, D. W., Rabiner, D. L., Kuhn, L., Pan, Y., & Sabet, R. F. (2018). Investigating teacher and student effects of the Incredible Years classroom management program in early elementary school. *Journal of School Psychology, 67*, 119-133. https://doi.org/10.1016/j.jsp.2017.10.004
S26*	Pérez-Escoda, N., Filella, G., Alegre, A., & Bisquerra, R. (2012). Developing the emotional competence of teachers and pupils in school contexts. *Electronic Journal of Research in Educational Psychology, 10*(3), 1183-1208. Retrieved from http://repositorio.ual.es/bitstream/handle/10835/1888/Art_28_756_eng.pdf?sequence=1
S26*	Pérez Escoda, N., Filella, G., Soldevila, A., & Fondevila, A. (2013). Evaluación de un programa de educación emocional para profesorado de primaria. *Educación XX1*, *16*, 233-254. https://doi.org/10.5944/educxx1.16.1.725
S27	Raver, C. C., Jones, S. M., Li-Grining, C. P., Metzger, M., Champion, K. M., & Sardin, L. (2008). Improving preschool classroom processes: Preliminary findings from a randomized trial implemented in Head Start settings. *Early childhood research quarterly, 23*(1), 10-26. https://doi.org/10.1016/j.ecresq.2007.09.001
S28*	Reiser, J. E., & McCarthy, C. J. (2018). Preliminary investigation of a stress prevention and mindfulness group for teachers. *The Journal for Specialists in Group Work, 43*(1), 2-34. https://doi.org/10.1080/01933922.2017.1338811
S29	Rivers, S. E., Brackett, M. A., Reyes, M. R., Elbertson, N. A., & Salovey, P. (2013). Improving the social and emotional climate of classrooms: A clustered randomized controlled trial testing the RULER approach. *Prevention science, 14*(1), 77-87. https://doi.org/10.1007/s11121-012-0305-2
S30*	Roberts, A. M., LoCasale-Crouch, J., Hamre, B. K., & Jamil, F. M. (2019). Preschool teachers' self-efficacy, burnout, and stress in online professional development: a mixed methods approach to understand change. *Journal of Early Childhood Teacher Education*, 1-22. https://doi.org/10.1080/10901027.2019.1638851
S31*	Roeser, R. W., Schonert-Reichl, K. A., Jha, A., Cullen, M., Wallace, L., Wilensky, R., … Harrison, J. (2013). Mindfulness training and reductions in teacher stress and burnout: Results from two randomized, waitlist-control field trials. *Journal of Educational Psychology*, *105*, 787-804. https://doi.org/10.1037/a0032093
S32*	Rupprecht, S., Paulus, P., & Walach, H. (2017). Mind the teachers! The impact of mindfulness training on self-regulation and classroom performance in a sample of German school teachers. *European Journal of Educational Research, 6*(4), 565-581. https://doi.org/10.12973/eu-jer.6.4.565
S34*	Talvio, M., Berg, M., & Lonka, K. (2015). How does continuing training on social interaction skills benefit teachers? *Procedia - Social and Behavioral Sciences, 171,* 820-829. https://doi.org/10.1016/j.sbspro.2015.01.197
S33*	Talvio, M., Lonka, K., Komulainen, E., Kuusela, M., & Lintunen, T. (2013). Revisiting Gordon's teacher effectiveness training: An intervention study on teachers' social and emotional learning. *Electronic Journal of Research in Educational Psychology, 11*(3), 693-716. https://doi.org/10.14204/ejrep.31.13073

S35*	Tarrasch, R., Berger, R., & Grossman, D. (2020). Mindfulness and compassion as key factors in improving teacher's well being. *Mindfulness*, 1-13. https://doi.org/10.1007/s12671-020-01304-x
S36*	Taylor, C., Harrison, J., Haimovitz, K., Oberle, E., Thomson, K., Schonert-Reichl, K., & Roeser, R. W. (2016). Examining ways that a mindfulness-based intervention reduces stress in public school teachers: A mixed-methods study. *Mindfulness*, *7*(1), 115-129. https://doi.org/10.1007/s12671-015-0425-4
S37*	Tsang, H. W., Cheung, W. M., Chan, A. H., Fung, K. M., Leung, A. Y., & Au, D. W. (2015). A pilot evaluation on a stress management programme using a combined approach of cognitive behavioural therapy (CBT) and complementary and alternative medicine (CAM) for elementary school teachers. *Stress and Health*, *31*(1), 35-43. https://doi.org/10.1002/smi.2522
S38	Webster-Stratton, C., Reid, M. J., & Stoolmiller, M. (2008). Preventing conduct problems and improving school readiness: Evaluation of the Incredible Years teacher and child training programs in high-risk schools. *Journal of Child Psychology and Psychiatry, 49*(5), 471-488. https://doi.org/10.1111/j.1469-7610.2007.01861.x
S39	Wills, H., Wehby, J., Caldarella, P., Kamps, D., & Romine, R. S. (2018). Classroom management that works: A replication trial of the CW-FIT program. *Exceptional Children, 84*(4), 437-456. https://doi.org/10.1177/0014402918771321

Note. Studies assessing SEL interventions which only targeted teachers have been marked with an asterisk. The remaining studies evaluated combined SEL interventions.

3 Coding Procedure

A set of 25 criteria was defined following literature recommendations on good practices (Gulamhussein, 2013; Higgins et al., 2011; Moher et al., 2015) to systematically document and organize information regarding each of the studies which met all the eligibility criteria and integrated the final pool of data. Criteria were grouped into four categories: Intervention; Sample; Formal training; Methodology and Results. Criteria concerning control of bias (Higgins et al., 2011) were included in the Methodology and Results category. Table S3 presents the full coding scheme with a detailed description of all the criteria used.

Table S3
Study coding scheme - description of the 25 criteria used

	**Criterion**	**Coding options**
Intervention	Intervention name	Name of the intervention / program used within the study
Intervention	Conceptual framework	Indicate the conceptual framework / frameworks underlying the development of the intervention / program used within the study (e.g., Emotional intelligence theory).
Intervention	Cross-session training	Did the intervention contemplate any training between formal sessions (e.g., homework assignments, tutoring sessions, ongoing coaching)?<br>YES or NO
Intervention	Target	Individual = targeted only teachers' SEL<br>Combined = targeted both teachers and students' SEL<br>*NOTE: Consider "combined" if students were trained, by their teacher, either at the same time or after the teacher training.*
Sample	N	List the sample size<br>*NOTE: Consider the total sample size considered for the descriptive analysis.*
Sample	Sex	List the percentage of female sample
Sample	Age	List mean age and standard deviation
Sample	Teaching experience	List mean of years of teaching experience and standard deviation
Sample	Urbanicity	List the type of area where schools were integrated: Urban, Sub-urban, Semi-rural, Rural.<br>*NOTE: If multiple sites were considered, list all.*

	Country	List the country where the study was developed.<br>*NOTE: If multiple countries were considered, list all.*
	Grade range	List the range of grades that participants taught.<br>When not explicitly stated, consider:<br>Pre-school: preK-K<br>Elementary school: 1-5<br>Middle/Secondary school: 6-8<br>High-school: 9-12<br>*NOTE: If multiple grades were considered, list all.*
Formal training	Dosage	Indicate the number of formal training hours.<br>*NOTE: Only include formal sessions, not home practice. When referring to "whole day sessions" consider 7-hour training, for "afternoon" or "half-day" consider 4-hour training. For combined studies, just include teacher training time.*
	Facilitator/Developer (F/D)	Was the person responsible for formal teacher training integrated in the original intervention development team?<br>YES or NO
	Developer/Researcher (D/R)	Are some of the authors of the study part of the original intervention development team?<br>YES or NO
	SEC addressed	List the social and emotional competencies specifically addressed by the intervention: Self-Awareness; Social-awareness; Self-management; Relationship skills; Responsible decision making.<br>*NOTE$_1$: If multiple were considered, list all.*<br>*NOTE$_2$: Presentation of the domains addressed may be directly specified on the paper or be inferred through the sessions' activities description and/or intervention aims (e.g., consider "social-awareness" if the authors describe as an aim "to understand others' feelings").*
	Fidelity assessment	List the type of measures used to assess the fidelity of the intervention: Observation, interviews and/or self-report.<br>*NOTE: List all that apply.*
	Fidelity report	Percentage of reported fidelity.<br>*NOTE: If multiple types were presented list all.*
Methodology and Results	Randomization (RCT; Control for selection bias)	Did the study use a randomized controlled trial design?<br>YES = If randomized controlled trial<br>YES – Cluster level = If randomization was at school-level<br>NO = If there was not randomization
	Blinding of participants and researchers (Control for performance bias)	Were there measures to control for performance bias?<br>YES or NO

Assessment times	List the assessment times considered within the study:<br>Pre-test, posttest and/or follow-up<br>*NOTE: List all that apply.*
Measures	List the type of measures used to assess outcomes:<br>Observation, behavioral, physiological and/or self-report<br>*NOTE: List all that apply.*
Outcomes	On which variables was the intervention's impact tested?<br>List the outcomes assessed in the study by domain:<br>SEC, Well-being, Psychological distress, Physical distress, and/or Classroom climate and teacher practices<br>*NOTE: List all that apply.*
Blinding of outcome assessment (Control for detection bias)	Were there measures to control for detection bias?<br>YES or NO
Incomplete outcome assessment (Control for attrition bias)	Whenever an incomplete outcome data was presented, did the study report the amount, nature and / or handling procedures for attrition and exclusion (e.g., missing or outlier analysis)?<br>YES or NO
Selective report (Control for reporting bias)	Whenever an incomplete outcome data was presented, did the study report how this selection process had been examined and what had been found?<br>YES or NO

4 Initial Results Regarding the Systematic Review

Table S4.1

Systematization of data from the studies eligible for the analysis regarding intervention and sample criteria

Study		Intervention			Sample							
**Code**	**Authors (year)**	**Name**	**Conceptual framework**	**Cross-session training**	**Target**	***N***	**Sex**	**Age**	**Teaching experience**	**Urbanicity**	**Country**	**Grade range**
S01	Ancona & Mendelson (2014)	Yoga and mindfulness intervention for teachers	n. s.	NO	IND	43	84.1	n. s.	8.8 (7.8)	URB	USA	1-8
S02	Benn et al. (2012)[1]	SMART-in-Education	n. s.	YES	IND	38	84.2	45.6 (n. s.)	n. s.	n. s.	USA	1-12
S03	Brown et al. (2010)	4Rs Program	n. s.	YES	COMB	82	93.9	36.4 (9.7)	7.0 (5.8)	URB	USA	3
S04	Carvalho et al. (2017)	Adapted MindUP	n. s.	YES	COMB	20	100	40.4 (6.30)	16.4 (5.4)	SUB	Portugal	3-4
S05	Castillo et al. (2013)	RULER (Teacher training component)	Emotional intelligence theory	NO	IND	47	70	44.3 (n. s.)	15.0 (9.7)	n. s.	Spain	K-8
S06	Castillo-Gualda et al. (2017)	RULER (Teacher training component)	Emotional intelligence theory	NO	IND	54	79.6	35.9 (8.45)	IG: 7.5 (4.5) CG: 9.1 (3.5)	URB	Spain	preK-8
S07	Conroy et al. (2015)	BEST in CLASS	n. s.	YES	COMB	53	100	n. s.	14.0 (n. s.)	n. s.	USA	preK-K
S08	Conroy et al. (2019)	BEST in CLASS	Behavioral and transactional theories	YES	COMB	186	97.3	n. s.	12.09 (n. s.)	n. s.	USA	preK-K
S09	Cook et al. (2017)	ACHIEVER Resilience Curriculum	Cognitive behavior therapy, Theory of planned behavior and Positive psychology	NO	IND	44	n. s.	n. s.	11.8 (4.2)	URB	USA	6-12
S10	Delgado et al. (2010)	Mindfulness & Human values Training	n. s.	NO	IND	31	93.6	n. s.	n. s.	URB	Spain	1-8
S11	Domitrovich et al. (2009)	PATHS curriculum	n. s.	NO	COMB	84	98.8	n. s.	n. s.	URB, RUR	USA	preK-K

	Bierman et al. (2013)					37	97.3					
S12	Domitrovich et al. (2016)	PATHS to PAX	n. s.	NO	COMB	350	87.7	n. s.	n. s.	URB	USA	K-5
S13	Figueiredo-Ferraz et al. (2013)	n. s.	Cognitive behavioral therapy	NO	IND	37	73.0	IG: 49.29 (n. s.) CG: 42.94 (n. s.)	n. s.	n. s.	Spain	1-4
S14	Flook et al. (2013)	Adapted MBSR	n. s.	YES	IND	18	88.9	43.1 (9.87)	12.8 (8.7)	n. s.	USA	1-4
S15	Frank et al. (2015)	Adapted MBSR	n. s.	YES	IND	36	77.8	40.7 (10.8)	n. s.	SUB	USA	9-12
S16	Gouda et al. (2016)[1]	Adapted MBSR	n. s.	YES	IND	29	n. s.	45.9 (8.5)	n. s.	n. s.	Germany	11
S17	Harris et al. (2016)	CALM	n. s.	NO	IND	64	88%	43.0 (12.5)	14.0 (9.0)	URB	USA	6-8
S18	Hickey et al. (2017)	IY-TCM	n. s.	YES	COMB	22	95.5	n. s.	n. s.	URB, SUB, RUR	Ireland	1-2
S19	Hutchings et al. (2013)	IY-TCM	n. s.	YES	COMB	12	100	34.8 (11.1)	9.3 (7.8)	URB, RUR	UK	K-2
S20	Jennings et al. (2013)	CARE	n. s.	YES	IND	53	89	36.0 (n. s.)	11.7 (n. s.)	URB, SUB	USA	preK-12
S21	Jennings et al. (2017)	CARE	n. s.	YES	IND	224	93	40.0 (n. s.)	10.6 (n. s.)	URB	USA	K-5
S22	Karimzadeh et al. (2014)	(EIT)	Bar-On theory	NO	IND	68	55.9	n. s.	n. s.	n. s.	Iran	4-5
S23	Kemeny et al. (2012)	Cultivating Emotional Balance Training	n. s.	NO	IND	82	100	41.1 (10.5)	n. s.	URB	USA	n. s.
S24	Morris et al. (2013)	Foundations of Learning	n. s.	NO	COMB	51	88.2	37.0 (9.3)	n. s.	URB	USA	K
S25	Murray et al. (2018)	IY-TCM	n. s.	NO	COMB	91	94.5	n. s.	10.9 (8.2)	SMR, RUR	USA	K-2

S26	Pérez-Escoda et al. (2012)[2]<br>Pérez-Escoda et al. (2013)	Emotional Education Program for Teachers	Emotional competence theoretical model	NO	IND	92	88	42.0 (n. s.)	n. s.	URB	Spain	1-4
S27	Raver et al. (2008)	IY-TCM	n. s.	NO	COMB	94	97	40.0 (11.0)	n. s.	URB	USA	preK-K
S28	Reiser & McCarthy (2018)	SPAM	Transactional model of stress and coping	YES	IND	45	78	31.0 (n. s.)	5.5 (n. s.)	n. s.	USA	6-12
S29	Rivers et al. (2013)<br>Hagelskamp et al. (2013)	RULER (Teacher training component + Feeling words curriculum)	Emotional intelligence theory	NO	COMB	105<br>90	70.5<br>91	n. s.	14.7 (10.0)	URB	USA	5-6
S30	Roberts et al. (2019)	Effective Classroom Interactions	n. s.	YES	IND	89	98	n. s.	8.6 (7.3)	n. s.	USA	preK-K
S31	Roeser et al. (2013)<br>Crain et al. (2017)	SMART-in-Education	n. s.	YES	IND	113	89	46.9 (9.2)	14.9	URB, SUB	Canada/ USA	1-8
S32	Rupprecht et al. (2017)	Adapted MBSR	n. s.	NO	IND	32	94	47.6 (9.7)	16.5 (10.9)	n. s.	Germany	n. s.
S33	Talvio et al. (2013)	TET	Gordon's theory of classroom management	NO	IND	69	n. s.	n. s.	n. s.	SMR	Finland	1-8
S34	Talvio et al. (2015)	n. s.	Gordon's theory of classroom management	NO	IND	40	IG: 85	IG: 37.0 (10.5)	IG: 4.5 (6.6)	n. s.	Finland	n. s.
S35	Tarrasch et al. (2020)	C2CIT	Psychology of attachment	YES	IND	44	90.9	IG: 34.9 (7.9) CG: 33.1 (7.6)	IG: 6.27 (7.99) CG: 7.05 (8.86)	n. s.	Israel	6-8
S36	Taylor et al. (2016)	SMART-in-Education	n. s.	NO	IND	59	89.8	47.0 (n. s.)	15.2 (n. s.)	URB	Canada	1-8
S37	Tsang et al. (2015)	Stress management program	Cognitive behavioral therapy	NO	IND	93	83.9	38.4 (8.5)	15.7 (8.5)	n. s.	China	1-5

S38	Webster-Stratton et al. (2008)	IY-TCM	Social cognitive learning theory	NO	COMB	153	95	n. s.	n. s.	SUB	USA	K-1
S39	Wills et al. (2018)	Class-Wide Function-Related Intervention Team	n. s.	NO	COMB	157	95.5	n. s.	n. s.	n. s.	USA	K-6

Note. [1] = data exclusively on teachers were analyzed; [2] = only data from study 1 was considered; *n. s.* = non specified; IND = individual intervention (i.e., targeted teachers only); COMB = combined intervention (i.e., targeted teachers and students); *N* = sample size; YT = young teachers; ET = experienced teachers; IG = intervention group; CG = control group; URB = urban schools; SUB = sub-urban schools; SMR = semi-rural schools; RUR = rural schools.

Table S4.2
Systematization of data from the studies eligible for the analysis regarding formal training criteria

**Study**		**Formal training**					
**Code**	**Authors (year)**	**Dose**	**Facilitator/Developer**	**Developer/Researcher**	**SEC address**	**Fidelity assessment**	**Fidelity report**
S01	Ancona & Mendelson (2014)	4.5h	YES	NO	SEA, SEM	n. s.	n. s.
S02	Benn et al. (2012)[1]	36h	NO	NO	SEA, SEM, SOA	OBS	High
S03	Brown et al. (2010)	25h	YES	NO	SOA, RS, RDM	n. s.	n. s.
S04	Carvalho et al. (2017)	50h	NO	NO	SEA, SEM, SOA, RS, RDM	n. s.	n. s.
S05	Castillo et al. (2013)	30h	NO	NO	SEA, SEM, SOA, RS, RDM	n. s.	n. s.
S06	Castillo-Gualda et al. (2017)	24h	NO	NO	SEA, SEM, SOA, RS, RDM	n. s.	n. s.
S07	Conroy et al. (2015)	34h	YES	YES	RS	n. s.	n. s.
S08	Conroy et al. (2019)	34h	YES	YES	SOA, RS	SR	85%
S09	Cook et al. (2017)	12.5h	YES	YES	SEA, SEM, SOA, RS, RDM	n. s.	n. s.
S10	Delgado et al. (2010)	30h	YES	YES	SEA, SEM	n. s.	n. s.
S11	Domitrovich et al. (2009)<br>Bierman et al. (2013)	28h	YES	YES	SOA, RS	SR	High
S12	Domitrovich et al. (2016)	25h	NO	YES	RS	n. s.	n. s.
S13	Figueiredo-Ferraz et al. (2013)	13h	YES	NO	SEA, SEM	n. s.	n. s.

S14	Flook et al. (2013)	26h	NO	YES	SEA, SEM, SOA, RS	n. s.	n. s.
S15	Frank et al. (2015)	16h	NO	NO	SEA, SEM, SOA, RS	n. s.	n. s.
S16	Gouda et al. (2016)[1]	23h	NO	NO	SEA, SEM, SOA, RS	n. s.	n. s.
S17	Harris et al. (2016)	21h	NO	YES	SEA, SEM	SR, OBS	94.3%
S18	Hickey et al. (2017)	35h	NO	NO	RS	SR	88%
S19	Hutchings et al. (2013)	35h	NO	NO	SEM, SOA, RS	n. s.	n. s.
S20	Jennings et al. (2013)	30h	YES	YES	SEA, SEM, SOA	SR, OBS	100%
S21	Jennings et al. (2017)	30h	NO	YES	SEA, SEM, SOA	OBS	88%
S22	Karimzadeh et al. (2014)	20h	YES	YES	SEA, SEM, SOA, RS, RDM	n. s.	n. s.
S23	Kemeny et al. (2012)	42h	NO	YES	SEA, SOA	n. s.	n. s.
S24	Morris et al. (2013)	30h	YES	YES	RS	n. s.	n. s.
S25	Murray et al. (2018)	35h	NO	NO	SEM, SOA, RS	OBS	85.2%
S26	Pérez-Escoda et al. (2012)[2]<br>Pérez-Escoda et al. (2013)	30h	YES	YES	SEA, SEM, SOA, RS, RDM	n. s.	n. s.
S27	Raver et al. (2008)	30h	NO	YES	SEM, SOA, RS	n. s.	n. s.
S28	Reiser & McCarthy (2018)	8h	YES	YES	SEA, SEM, RS	n. s.	n. s.
S29	Rivers et al. (2013)	18h	YES	YES	SEA, SEM, SOA, RS, RDM	n. s.	n. s.

	Hagelskamp et al. (2013)						
S30	Roberts et al. (2019)	35h	YES	YES	SEA, SOA, RS	SR	n. s.
S31	Roeser et al. (2013)<br>Crain et al. (2017)	36h	NO	NO	SEA, SEM, SOA	n. s.	n. s.
S32	Rupprecht et al. (2017)	26h	NO	NO	SEA, SEM, SOA, RS	n. s.	n. s.
S33	Talvio et al. (2013)	28h	NO	NO	SEA, SEM	n. s.	n. s.
S34	Talvio et al. (2015)	20h	YES	YES	SEA, SOA, RDM	n. s.	n. s.
S35	Tarrasch et al. (2020)	30h	YES	YES	SEA, SEM, SOA, RS	n. s.	n. s.
S36	Taylor et al. (2016)	36h	NO	NO	SEA, SEM, SOA	n. s.	n. s.
S37	Tsang et al. (2015)	12h	YES	YES	SEA, SEM	n. s.	n. s.
S38	Webster-Stratton et al. (2008)	28h	YES	YES	SOA, RS, RDM	n. s.	n. s.
S39	Wills et al. (2018)	2h	YES	YES	SEM, SOA, RS	n. s.	n. s.

Note. [1] = only data exclusively on teachers were analyzed; [2] = only data from study 1 was considered; *n. s.* = non specified; SEA = self-awareness; SOA= social-awareness; SEM = self-management; RS = relationship skills; RDM = responsible decision making.

Table S4.3
Systematization of data from the studies eligible for the analysis regarding methodology and results criteria

**Study**		**Methodology and Results**							
**Code**	**Authors**	**RCT**	**Blinding of participants and researchers**	**Assessment times**	**Measures**	**Outcomes**	**Blinding of outcome assessment**	**Incomplete outcome assessment**	**Selective report**
S01	Ancona & Mendelson (2014)	YES - Cluster level	NO	T1, T2	SR	DISTRESS	NO	NO	n. a.
S02	Benn et al. (2012)[1]	YES	n. s.	T1, T2, T3	SR	SEC, WELL	n. s.	YES	NO
S03	Brown et al. (2010)	YES - Cluster level	NO	T1, T2	OBS	CLASS	YES	YES	n. a.
S04	Carvalho et al. (2017)	YES	NO	T1, T2	SR	DISTRESS, SEC, WELL	n. s.	n. s.	n. a.
S05	Castillo et al. (2013)	NO	NO	T1, T2	SR	CLASS, DISTRESS, WELL	NO	n. s.	n. a.
S06	Castillo-Gualda et al. (2017)	YES - Cluster level	NO	T1, T2	SR	DISTRESS, SEC, WELL	n. s.	n. s.	n. a.
S07	Conroy et al. (2015)	YES	NO	T1, T2, T3	OBS	CLASS	n. s.	n. s.	n. a.
S08	Conroy et al. (2019)	YES	NO	T1, T2	SR, OBS	CLASS, WELL	n. s.	YES	n. a.
S09	Cook et al. (2017)	YES	n. s.	T1, T2	SR	DISTRESS, WELL	n. s.	n. s.	n. a.
S10	Delgado et al. (2010)	NO	NO	T1, T2	SR, BH, PI	DISTRESS, PHYS, SEC	n. s.	n. s.	n. a.
S11	Domitrovich et al. (2009)<br>Bierman et al. (2013)	YES	YES	T1, T2, T3	OBS	CLASS	YES	YES	n. a.
S12	Domitrovich et al. (2016)	YES	n. s.	T1, T2	SR	DISTRESS, SEC, WELL	n. s.	YES	n. a.
S13	Figueiredo-Ferraz et al. (2013)	NO	NO	T1, T2	SR, PI	DISTRESS, PHYS	NO	n. s.	n. a.
S14	Flook et al. (2013)	YES	n. s.	T1, T2	SR, OBS, PI	CLASS, DISTRESS, PHYS, SEC, WELL	n. s.	YES	n. a.
S15	Frank et al. (2015)	NO	n. s.	T1, T2	SR	DISTRESS, SEC, PHYS, WELL	n. s.	YES	n. a.
S16	Gouda et al. (2016)[1]	NO	n. s.	T1, T2	SR	DISTRESS, SEC, WELL	YES	n. s.	n. a.
S17	Harris et al. (2016)	YES - Cluster level	n. s.	T1, T2	SR, PI	DISTRESS, PHYS, SEC, WELL	n. s.	n. s.	n. a.
S18	Hickey et al. (2017)	YES	YES	T1, T2	SR, OBS	CLASS	Partial	n. s.	n. a.

S19	Hutchings et al. (2013)	YES - Cluster level	NO	T1, T2	SR, OBS	CLASS	NO	YES	n. a.
S20	Jennings et al. (2013)	YES	NO	T1, T2	SR	DISTRESS, PHYS, SEC, WELL	n. s.	YES	n. a.
S21	Jennings et al. (2017)	YES	NO	T1, T2	SR, OBS	CLASS, DISTRESS, PHYS, SEC, WELL	YES	YES	n. a.
S22	Karimzadeh et al. (2014)	YES	n. s.	T1, T2, T3	SR	SEC, WELL	n. s.	n. s.	n. a.
S23	Kemeny et al. (2012)	YES	NO	T1, T2	SR, BH, PI	DISTRESS, PHYS, SEC, WELL	YES	n. s.	n. a.
S24	Morris et al. (2013)	YES - Cluster level	NO	T1, T2	OBS	CLASS	NO	YES	n. a.
S25	Murray et al. (2018)	YES	NO	T1, T2, T3	OBS	CLASS	YES	YES	n. a.
S26	Pérez-Escoda et al. (2012)[2]<br>Pérez-Escoda et al. (2013)	NO	NO	T1, T2	SR	DISTRESS, SEC, WELL	n. s.	n. s.	n. a.
S27	Raver et al. (2008)	YES - Cluster level	NO	T1, T2	OBS	CLASS	YES	YES	n. a.
S28	Reiser & McCarthy (2018)	NO	NO	T1, T2	SR	DISTRESS, SEC, WELL	NO	n. s.	n. a.
S29	Rivers et al. (2013)<br>Hagelskamp et al. (2013)	YES - Cluster level	NO	T1, T2, T3	SR, OBS	CLASS	YES	YES	n. a.
S30	Roberts et al. (2019)	YES	n. s.	T1, T2	SR	DISTRESS, WELL	YES	YES	n. a.
S31	Roeser et al. (2013)<br>Crain et al. (2017)	YES	NO	T1, T2, T3	SR, BH, PI	DISTRESS, PHYS, SEC, WELL	n. s.	n. s.	n. a.
S32	Rupprecht et al. (2017)	NO	n. s.	T1, T2, T3	SR	DISTRESS, PHYS, SEC, WELL	n. s.	n. s.	n. a.
S33	Talvio et al. (2013)	NO	n. s.	T1, T2	SR	CLASS, SEC, WELL	n. s.	n. s.	n. a.
S34	Talvio et al. (2015)	NO	n. s.	T1, T2	SR	CLASS	n. s.	n. s.	n. a.
S35	Tarrasch et al. (2020)	YES	n. s.	T1, T2	SR	DISTRESS, SEC, WELL	n. s.	n. s.	n. a.
S36	Taylor et al. (2016)	YES	n. s.	T1, T2, T3	SR	DISTRESS, SEC, WELL	YES	n. s.	n. a.
S37	Tsang et al. (2015)	NO	NO	T1, T2, T3	SR, PI	DISTRESS, PHYS, WELL	YES	YES	n. a.
S38	Webster-Stratton et al. (2008)	YES - Cluster level	NO	T1, T2	OBS	CLASS	YES	YES	n. a.

S39	Wills et al. (2018)	YES - Cluster level	n. s.	T1, T2	OBS	CLASS	n. s.	n. s.	n. a.

Note. [1] = only data exclusively on teachers were analyzed; [2] = only data from study 1 was considered; *n. s.* = non specified; *n. a.* = not applicable; T1 = Pre-test; T2 = Posttest; T3 = Follow-up; SR = self-report measures; HR = hetero-report measures; BH = behavioral measures (task realization); OBS = observational measures; PI = physiological indicators; CLASS = classroom climate and instructional practices; SEC = social and emotional competence; DISTRESS = psychological distress; PHYS = physical distress; WELL = well-being.

4.1 Funnel Plots Which Help Informing Possible Publication Bias

Figure S1
Funnel plot for pooled effects

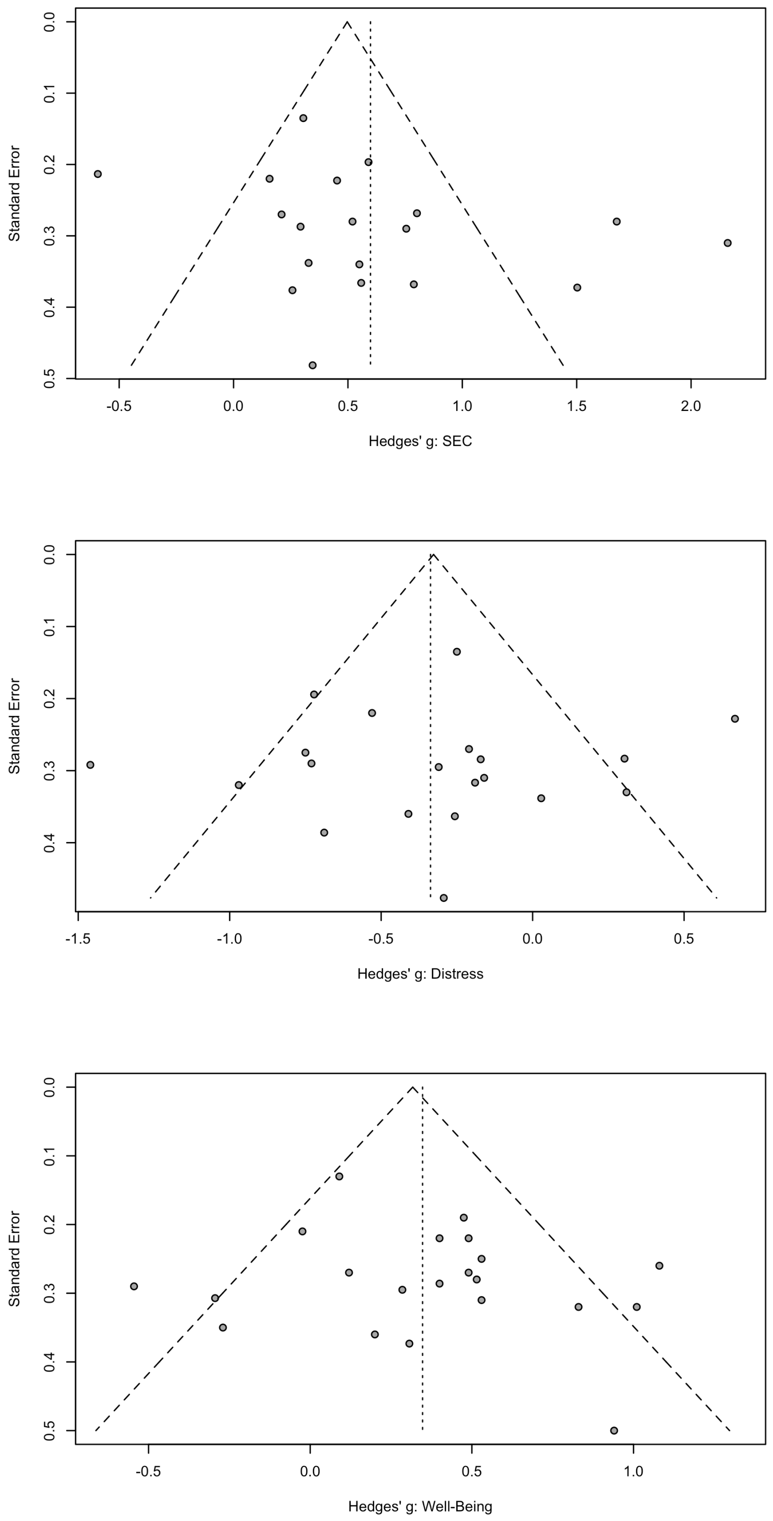

Supplement: Supplementary file 1 [file Data_Sheet_1.pdf]
